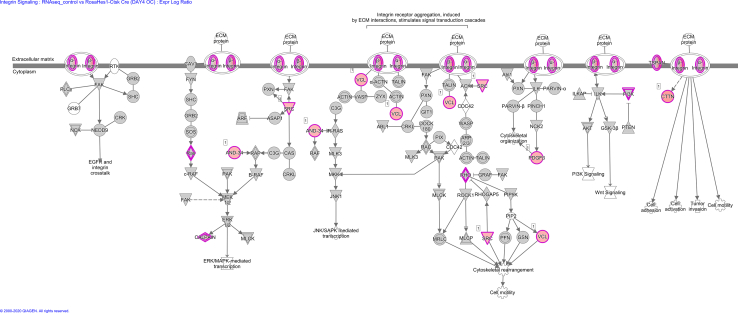# Hairy and enhancer of split 1 is a primary effector of NOTCH2 signaling and induces osteoclast differentiation and function

**DOI:** 10.1016/j.jbc.2021.101376

**Published:** 2021-11-03

**Authors:** Jungeun Yu, Lauren Schilling, Tabitha Eller, Ernesto Canalis

**Affiliations:** 1 Department of Orthopaedic Surgery, UConn Health, Farmington, Connecticut, USA; 2 UConn Musculoskeletal Institute, UConn Health, Farmington, Connecticut, USA; 3 Department of Medicine, UConn Health, Farmington, Connecticut, USA

**Keywords:** HES1, bone resorption, bone remodeling, osteoclasts, Hajdu–Cheney syndrome, NOTCH2, α-MEM, α-minimum essential medium, μCT, microcomputed tomography, Ad-Cre, adenoviruses carrying cytomegalovirus-Cre, Ad-GFP, adenoviruses carrying GFP, *Blimp1*, B lymphocyte–induced maturation protein 1, BMM, bone marrow–derived macrophage, BV/TV, bone volume/total volume, cDNA, complementary DNA, FBS, fetal bovine serum, HCS, Hajdu–Cheney syndrome, HES1, hairy and enhancer of split 1, IL, interleukin, IPA, ingenuity pathway analysis, M-CSF, macrophage colony-stimulating factor, NFATc1, nuclear factor of activated T cells, cytoplasmic 1, RANKL, receptor activator of NF-κB ligand, SMI, structure model index, TNFα, tumor necrosis factor α, TRAP, tartrate resistant acid phosphatase

## Abstract

*Notch2*^*tm1.1Ecan*^ mice, which harbor a mutation replicating that found in Hajdu–Cheney syndrome, exhibit marked osteopenia because of increased osteoclast number and bone resorption. Hairy and enhancer of split 1 (HES1) is a Notch target gene and a transcriptional modulator that determines osteoclast cell fate decisions. Transcript levels of *Hes1* increase in *Notch2*^*tm1.1Ecan*^ bone marrow–derived macrophages (BMMs) as they mature into osteoclasts, suggesting a role in osteoclastogenesis. To determine whether HES1 is responsible for the phenotype of *Notch2*^*tm1.1Ecan*^ mice and the skeletal manifestations of Hajdu–Cheney syndrome, *Hes1* was inactivated in *Ctsk*-expressing cells from *Notch2*^*tm1.1Ecan*^ mice. *Ctsk* encodes the protease cathepsin K, which is expressed preferentially by osteoclasts. We found that the osteopenia of *Notch2*^*tm1.1Ecan*^ mice was ameliorated, and the enhanced osteoclastogenesis was reversed in the context of the *Hes1* inactivation. Microcomputed tomography revealed that the downregulation of *Hes1* in *Ctsk*-expressing cells led to increased bone volume/total volume in female mice. In addition, cultures of BMMs from *Ctsk*^*Cre/WT*^;*Hes1*^*Δ/Δ*^ mice displayed a decrease in osteoclast number and size and decreased bone-resorbing capacity. Moreover, activation of HES1 in *Ctsk*-expressing cells led to osteopenia and enhanced osteoclast number, size, and bone resorptive capacity in BMM cultures. Osteoclast phenotypes and RNA-Seq of cells in which HES1 was activated revealed that HES1 modulates cell–cell fusion and bone-resorbing capacity by supporting sealing zone formation. In conclusion, we demonstrate that HES1 is mechanistically relevant to the skeletal manifestation of *Notch2*^*tm1.1Ecan*^ mice and is a novel determinant of osteoclast differentiation and function.

Osteoclasts are multinucleated giant cells that are responsible for bone resorption and essential to maintain bone homeostasis. Osteoclasts are derived from the differentiation and fusion of mononuclear cells of the myeloid lineage by the actions of macrophage colony-stimulating factor (M-CSF) and receptor activator of NF-κB ligand (RANKL) (1, 2). RANKL triggers downstream signaling to induce the expression of transcription factors required for osteoclastogenesis, such as nuclear factor of activated T cells, cytoplasmic 1 (NFATc1) (3, 4, 5, 6). An imbalance of physiological or pathological conditions causing dysregulation of osteoclast differentiation and function leads to diseases associated with alterations in bone mass (7, 8).

Hajdu–Cheney syndrome (HCS) (Online Mendelian Inheritance in Man: 102500) is a rare and devastating disorder characterized by numerous skeletal manifestations, including craniofacial developmental defects, short stature, bone loss with fractures, and acroosteolysis associated with inflammation of the distal phalanges (9, 10, 11, 12). HCS is associated with mutations or short deletions in exon 34 of *NOTCH2* upstream of the PEST domain, which is required for the ubiquitination and degradation of NOTCH2 (12, 13, 14, 15, 16). The HCS pathogenic variants lead to the premature termination of a protein product lacking sequences necessary for the proteasomal degradation of the NOTCH2 intracellular domain so that the protein is stable and a gain-of-NOTCH2 function ensues. Autosomal dominant inheritance as well as *de novo* heterozygous mutations have been reported (12, 13, 14, 15, 16).

Our laboratory created a knock-in mouse model harboring a *Notch2*^*6955C>T*^ mutation reproducing HCS and termed *Notch2*^*tm1.1Ecan*^ (also known as *Notch2*^*Q2319X*^) (17, 18). The homozygous mutation is associated with craniofacial developmental abnormalities and is lethal, and heterozygous *Notch2*^*tm1.1Ecan*^ mutant mice exhibit profound osteopenia and short limbs, reproducing functional outcomes of the human disease and establishing the first model for the study of HCS (12, 17). *Notch2*^*tm1.1Ecan*^ mice have increased bone resorption secondary to a direct effect of the gain-of-NOTCH2 function on osteoclastogenesis as well as the increased expression of RANKL by cells of the osteoblast lineage (17). These are unique functional properties of NOTCH2, which are distinct from those reported for other Notch receptors (19, 20). Indeed, NOTCH1 inhibits osteoclastogenesis directly, and NOTCH3 is not expressed in the myeloid lineage; although, by inducing RANKL in cells of the osteoblast lineage, it enhances osteoclastogenesis indirectly (21, 22, 23). Low levels of NOTCH4 are expressed in the myeloid lineage, and it is not known to play a role in osteoblastogenesis (24).

Cultures of bone marrow macrophages (BMMs) revealed that the expression of *Hes1*, a Notch target gene, is enhanced as cells mature as osteoclasts, and the increased expression is of greater magnitude in cultures from *Notch2*^*tm1.1Ecan*^ mice (17, 25). Importantly, other Notch target genes, such as *Hes3*, *Hes5*, *Hey1*, *Hey2*, and *HeyL*, are either expressed at very low levels or not detected in BMMs from control or mutant mice. This observation suggests that hairy and enhancer of split 1 (HES1) may be an important regulator of osteoclastogenesis and is in part responsible for the HCS phenotype.

HES1 is a transcriptional modulator that plays a role in the differentiation of embryonic stem and mesenchymal cells (26, 27). Although HES1 is considered a transcriptional repressor, transcription factors can function as either positive or negative regulators of transcription in a cell context–dependent manner (28, 29). In addition, calcium/calmodulin-dependent protein kinase 2 can convert HES1 from a repressor to an activator of transcription (30, 31). Misexpression of *Hes1* in the osteoblast lineage has demonstrated a role as an inhibitor of osteoblast differentiation and function (32). The role of HES1 in osteoclastogenesis is unknown.

The intent of the present study was to determine whether HES1 was mechanistically relevant to the HCS phenotype and to define the function of HES1 in osteoclast differentiation *in vitro* and *in vivo*. For this purpose, *Hes1* was induced or inactivated in *Ctsk*-expressing cells of the osteoclast lineage. To determine whether HES1 had a mechanistic role in the skeletal phenotype of HCS, *Notch2*^*tm1.1Ecan*^ mice were studied in the context of the *Hes1* inactivation in *Ctsk*-expressing cells. Skeletal phenotypes were determined by microcomputed tomography (μCT) and histomorphometry and cellular effects by the study of osteoclast differentiation and resorption activity *in vitro*.

## Results

### Inactivation of Hes1 reverses the effect of the Hajdu–Cheney mutation on osteoclastogenesis

**Figure 1:**
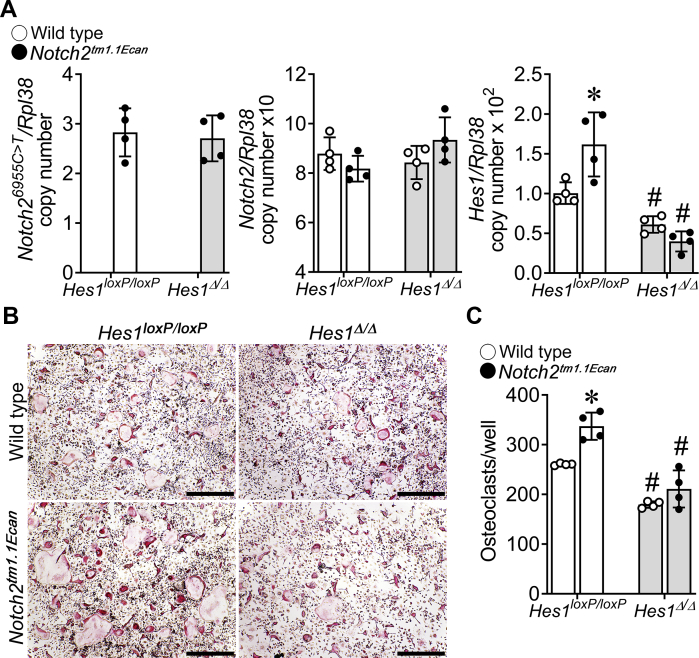
***Hes1* inactivation reverses the effect of the Hajdu–Cheney mutation on osteoclastogenesis.** BMMs derived from 2-month-old *Notch2*^*tm1.1Ecan*^*;Hes1*^*loxP/loxP*^ and *Hes1*^*loxP/loxP*^ littermate controls were cultured for 2 days with M-CSF at 30 ng/ml and RANKL at 10 ng/ml and transduced with adenoviruses carrying CMV-Cre (Ad-Cre) or adenoviruses carrying GFP (Ad-GFP) as control at MOI 100 and cultured for two additional days in the presence of M-CSF at 30 ng/ml and RANKL at 10 ng/ml until the formation of multinucleated TRAP-positive cells. *A*, total RNA was extracted, and gene expression was determined by quantitative RT–PCR. Data are expressed as *Notch2*^*6955C>T*^, *Notch2*, and *Hes1*, corrected for *Rpl38* copy number. *B*, representative images of TRAP-stained multinucleated cells are shown. The scale bars in the *right corner* represent 500 μm. *C*, TRAP-positive cells with more than three nuclei were considered osteoclasts and counted. Values are means ± SD; n = 4 technical replicates for WT (*open circles*) and *Notch2*^*tm1.1Ecan*^ (*closed circles*) cells in the context of *Hes1*^*loxP/loxP*^ (*white bar*) or *Hes1*^*Δ/Δ*^ (*gray bar*) deleted alleles. Representative data are shown from two independent experiments. ∗Significantly different between *Notch2*^*tm1.1Ecan*^ and control, *p* < 0.05. #Significantly different between *Hes1*^*Δ/Δ*^ and *Hes1*^*loxP/loxP*^, *p* < 0.05. BMM, bone marrow–derived macrophage; M-CSF, macrophage colony-stimulating factor; MOI, multiplicity of infection; RANKL, receptor activator of NF-κB ligand; TRAP, tartrate resistant acid phosphatase.

To determine whether HES1 played a role in the enhanced osteoclastogenesis observed in *Notch2*^*tm1.1Ecan*^ mice, osteoclast precursors from *Notch2*^*tm1.1Ecan*^*;Hes1*^*loxP/loxP*^ and *Hes1*^*loxP/loxP*^ littermate controls were transduced with adenoviruses carrying cytomegalovirus-Cre (Ad-Cre) or GFP (Ad-GFP) control vectors. *Hes1* mRNA levels were increased in *Notch2*^*tm1.1Ecan*^ cells and decreased significantly in *Notch2*^*tm1.1Ecan*^*;Hes1*^*Δ/Δ*^ and *Hes1*^*Δ/Δ*^ osteoclasts transduced with Ad-Cre compared with *Notch2*^*tm1.1Ecan*^*;Hes1*^*loxP/loxP*^ and *Hes1*^*loxP/loxP*^ cells transduced with Ad-GFP. *Notch2* and *Notch2*^*6955C>T*^ mutant transcripts were not affected by the *Hes1* inactivation (Fig. 1). *Notch2*^*tm1.1Ecan*^*;Hes1*^*loxP/loxP*^ osteoclast precursors treated with RANKL exhibited an increase in osteoclast number compared with *Hes1*^*loxP/loxP*^ cells. Osteoclast number was decreased significantly in *Notch2*^*tm1.1Ecan*^; *Hes1*^*Δ/Δ*^ and *Hes1*^*Δ/Δ*^ cells so that the *Hes1* inactivation reversed the enhanced osteoclastogenesis observed in the context of the *Notch2*^*tm1.1Ecan*^ mutation. In addition, basal levels of osteoclastogenesis were reduced in *Hes1*^*Δ/Δ*^ cells, suggesting a role of HES1 in osteoclastogenesis under physiological conditions (Fig. 1).

**Figure 2:**
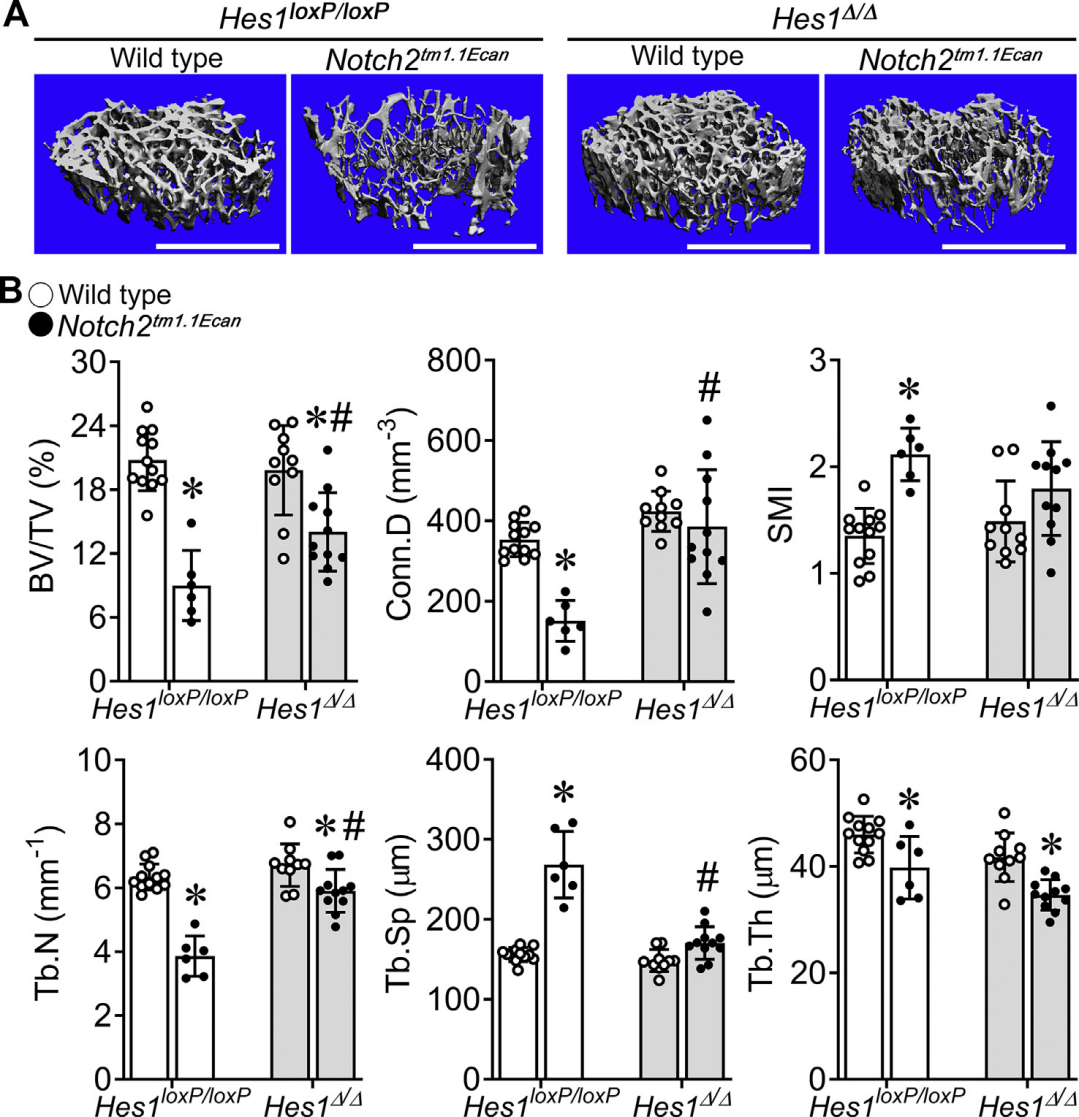
***Hes1* inactivation in *Ctsk*-expressing cells ameliorates the osteopenia of the Hajdu–Cheney mutation.** μCT was performed on 2-month-old WT (*open circles*) or *Notch2*^*tm1.1Ecan*^ (*closed circles*) mice in a *Hes1*^*Δ/Δ*^ (*gray bar*) or *Hes1*^*loxP/loxP*^ (*white bar*) genetic background by crossing *Ctsk*^*Cre/WT*^*;Hes1*^*loxP/loxP*^ with *Notch2*^*tm1.1Ecan*^*;Hes1*^*loxP/loxP*^ mice. *A*, representative images show osteopenic cancellous bone of the distal femur in *Notch2*^*tm1.1Ecan*^*;Hes1*^*loxP/loxP*^ male mice and its amelioration by the *Hes1* inactivation. The scale bar in the *right corner* represents 1 mm. *B*, parameters shown are bone volume/total volume (BV/TV, %); connectivity density (Conn.D, mm^−3^); structure model index (SMI); trabecular number (Tb.N, mm^−1^), trabecular separation (Tb.Sp, μm), and trabecular thickness (Tb.Th, μm). Values are means ± SD; n = 12 for control *Hes1*^*loxP/loxP*^ and n = 6 for *Notch2*^*tm1.1Ecan*^*;Hes1*^*loxP/loxP*^; n = 10 for *Ctsk*^*Cre/WT*^*;Hes1*^*Δ/Δ*^ and n = 11 for *Ctsk*^*Cre/WT*^*;Notch2*^*tm1.1Ecan*^*;Hes1*^*Δ/Δ*^. ∗Significantly different between *Notch2*^*tm1.1Ecan*^ and control, *p* < 0.05. #Significantly different between *Hes1*^*Δ/Δ*^ and *Hes1*^*loxP/loxP*^, *p* < 0.05. μCT, microcomputed tomography.

**Supplemental Figure S1:**
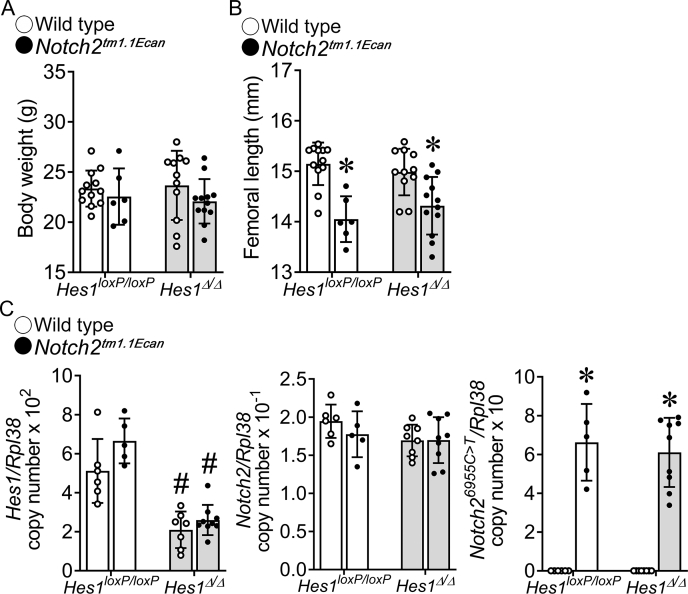


To determine whether the inactivation of *Hes1* could reverse the osteopenia of the *Notch2*^*tm1.1Ecan*^ mutation, *Ctsk*^*Cre/WT*^*;Hes1*^*loxP/loxP*^ mice were crossed with *Notch2*^*tm1.1Ecan*^*;Hes1*^*loxP/loxP*^ mice to inactivate *Hes1* in the context of the *Notch2* mutation. The transcript levels of *Hes1* were decreased in bone extracts from 2-month-old male *Ctsk*^*Cre/WT*^*;Hes1*^*Δ/Δ*^ and *Ctsk*^*Cre/WT*^*;Notch2*^*tm1.1Ecan*^*;Hes1*^*Δ/Δ*^ mice compared with control, whereas *Notch2* WT and mutant (*Notch2*^*6955C>T*^) mRNA levels were not affected (Fig. S1). Confirming prior observations, *Notch2*^*tm1.1Ecan*^ mice displayed cancellous bone osteopenia associated with decreased connectivity and trabecular number (Fig. 2). The *Hes1* inactivation by itself did not alter bone microarchitectural parameters in 2-month-old male mice compared with control sex-matched WT mice. The decreased cancellous bone volume/total volume (BV/TV) observed in *Notch2*^*tm1.1Ecan*^ was significantly increased in the context of the *Hes1* inactivation associated with increased connectivity and trabecular number so that the osteopenia of *Notch2*^*tm1.1Ecan*^ mice was ameliorated in *Ctsk*^*Cre/WT*^*;Notch2*^*tm1.1Ecan*^*;Hes1*^*Δ/Δ*^ mice (Fig. 2). Cancellous bone histomorphometry confirmed previous work and demonstrated an increase in osteoclast number and bone resorption, without an effect on osteoblast number and bone formation, in *Notch2*^*tm1.1Ecan*^ mice (17). The increased osteoclast number and eroded surface found in *Notch2*^*tm1.1Ecan*^ mice were decreased ∼50% in the context of the *Hes1* inactivation, so that both parameters were no longer increased in *Ctsk*^*Cre/WT*^*;Notch2*^*tm1.1Ecan*^*;Hes1*^*Δ/Δ*^ male mice compared with *Notch2*^*tm1.1Ecan*^*;Hes*^*loxP/loxP*^ control mice (Table 1). These results indicate that HES1 is mechanistically relevant to the osteopenia of *Notch2*^*tm1.1Ecan*^ mice although they suggest a minor role of HES1 in the bone architecture of male mice. The *Hes1* deletion had only a modest effect on the cortical osteopenic phenotype (not shown) and did not affect the decrease in femoral length observed in *Notch2*^*tm1.1Ecan*^ mice (Fig. S1).

**Table 1:** Cancellous bone histomorphometry of 2-month-old *Hes1*^*loxP/loxP*^, *Notch2*^*tm1.1Ecan*^*;Hes1*^*loxP/loxP*^, *Ctsk*^*Cre/WT*^*;Hes1*^*Δ/Δ*^ and *Ctsk*^*Cre/WT*^*;Notch2*^*tm1.1Ecan*^*;Hes1*^*Δ/Δ*^ male mice

Distal femur trabecular bone	*Hes1*^*loxP/loxP*^		*Hes1*^*Δ/Δ*^	
	WT	*Notch2*^*tm1.1Ecan*^	WT	*Notch2*^*tm1.1Ecan*^
	n = 4			
BV/TV (%)	32.1 ± 2.8	14.2 ± 3.6^a^	37.4 ± 1.9	24.9 ± 7.4^a^^,^^b^
Trabecular separation (μm)	117 ± 5	236 ± 53^a^	94 ± 7	130 ± 40^b^
Trabecular number (1/mm)	5.8 ± 0.1	3.9 ± 1.0^a^	6.7 ± 0.5	6.0 ± 1.2^b^
Trabecular thickness (μm)	55 ± 5	39 ± 4^a^	56 ± 5	41 ± 6^a^
Osteoblast surface/bone surface (%)	14.7 ± 0.9	19 ± 1.3	13 ± 1.9	18 ± 4.3
Osteoblasts/bone perimeter (1/mm)	9.1 ± 0.9	11.3 ± 0.6	8.5 ± 1.0	10.1 ± 1.6
Osteoclast surface/bone surface (%)	10.4 ± 3.7	20.1 ± 3.4^a^	12.1 ± 2.2	9.1 ± 4.1^b^
Osteoclasts/bone perimeter (1/mm)	3.2 ± 1.3	6.1 ± 1.2^a^	3.6 ± 0.8	2.8 ± 1.7^b^
Eroded surface/bone surface (%)	10.3 ± 1.3	13.4 ± 2.2^a^	11.9 ± 0.8	7.1 ± 1.1^a^^,^^b^
Mineral apposition rate (μm/day)	3.3 ± 1.0	2.8 ± 0.2	2.6 ± 0.1	2.4 ± 0.2
Mineralizing surface/bone surface (%)	31.2 ± 2.8	27.7 ± 3.5	35.1 ± 4.4	32.3 ± 2.3
Bone formation rate (μm^3^/μm^2^/day)	1.0 ± 0.3	0.8 ± 0.1	0.9 ± 0.1	0.8 ± 0.1

Bone histomorphometry was performed on distal femurs from 2-month-old *Hes1*^*loxP/loxP*^, *Notch2*^*tm1.1Ecan*^*;Hes1*^*loxP/loxP*^, *Ctsk*^*Cre/WT*^*;Hes1*^*Δ/Δ*^, and *Ctsk*^*Cre/WT*^;*Notch2*^*tm1.1Ecan*^;*Hes1*^*Δ/Δ*^ male mice. Values are means ± SD.

a Significantly different between *Notch2*^*tm1.1Ecan*^ and WT, *p* < 0.05.

b Significantly different between *Hes1*^*Δ/Δ*^ and *Hes1*^*loxP/loxP*^, *p* < 0.05.

### HES1 is a determinant of osteoclastogenesis *in vitro*

**Figure 3:**
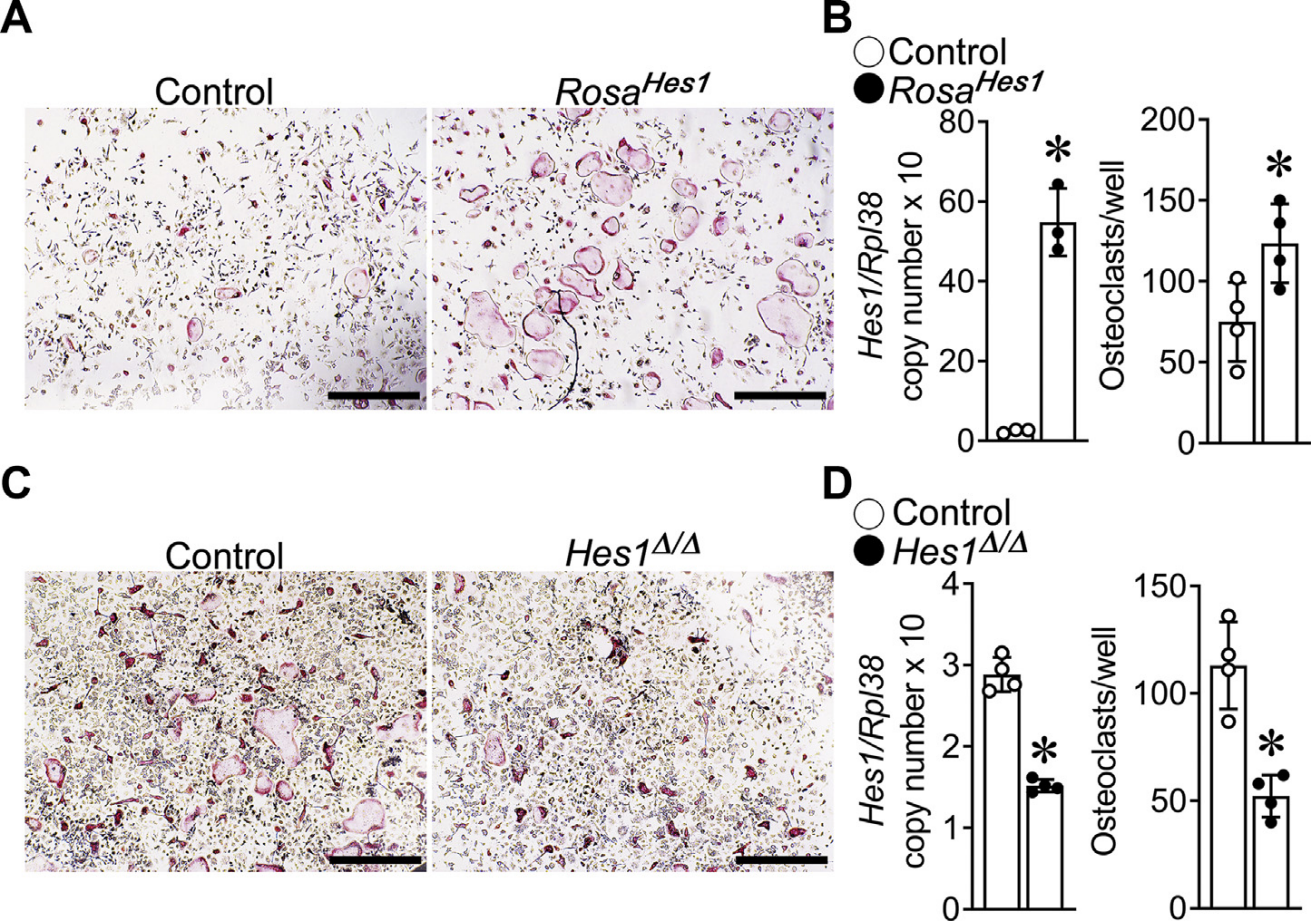
**HES1 is required for osteoclastogenesis *in vitro*.** BMMs derived from 2-month-old *Rosa*^*Hes1*^ (*A* and *B*) or *Hes*^*loxP/loxP*^ mice (*C* and *D*) were cultured in the presence of M-CSF at 30 ng/ml and RANKL at 10 ng/ml for 2 days. Cells were transduced with Ad-Cre (*closed circles*), to recombine *loxP* flanked sequences, or Ad-GFP (*open circles*) as a control and then cultured for two additional days. *A* and *C*, representative images of TRAP-stained multinucleated cells are shown. The scale bar in the *right corner* represents 500 μm. *B* and *D*, *Hes1* transcript levels were measured by quantitative RT–PCR in total RNA from osteoclasts. Transcript levels are reported as copy number corrected for *Rpl38* (*left*). TRAP-positive cells with more than three nuclei were considered osteoclasts (*right*). Values are means ± SD; n = 3 or 4 technical replicates for control (*open circles*) and either *Hes1*^*Δ/Δ*^ or recombined *Rosa*^*Hes1*^ (*closed circles*) cells. Representative data are shown from two independent experiments. ∗Significantly different between *Hes1*^*Δ/Δ*^ and control, *p* < 0.05; or recombined *Rosa*^*Hes1*^ and control, *p* < 0.05. BMM, bone marrow–derived macrophage; HES1, hairy and enhancer of split 1; M-CSF, macrophage colony-stimulating factor; RANKL, receptor activator of NF-κB ligand; TRAP, tartrate resistant acid phosphatase.

To ascertain the function of HES1 in cells of the osteoclast lineage, BMMs from either *Hes1*^*loxP/loxP*^ or *Rosa*^*[STOP]Hes1*^ mice were isolated. BMMs were cultured in the presence of M-CSF and RANKL for 2 days and transduced with Ad-Cre to delete *loxP* flanked sequences or Ad-GFP as control. Excision of the STOP cassette in *Rosa*^*Hes1*^ cells resulted in a 20-fold induction of *Hes1* mRNA and a 1.7-fold increase in osteoclastogenesis compared with *Rosa*^*[STOP]Hes1*^ cultures transduced with Ad-GFP (Fig. 3). Conversely, deletion of *Hes1* resulted in a 50% reduction in *Hes1* mRNA levels and a 50% decrease in osteoclast number compared with control cultures transduced with Ad-GFP (Fig. 3). The results demonstrate that HES1 is a determinant of osteoclast differentiation *in vitro*.

### Inactivation of Hes1 in osteoclasts of female mice increases BV *in vivo*

**Figure 4:**
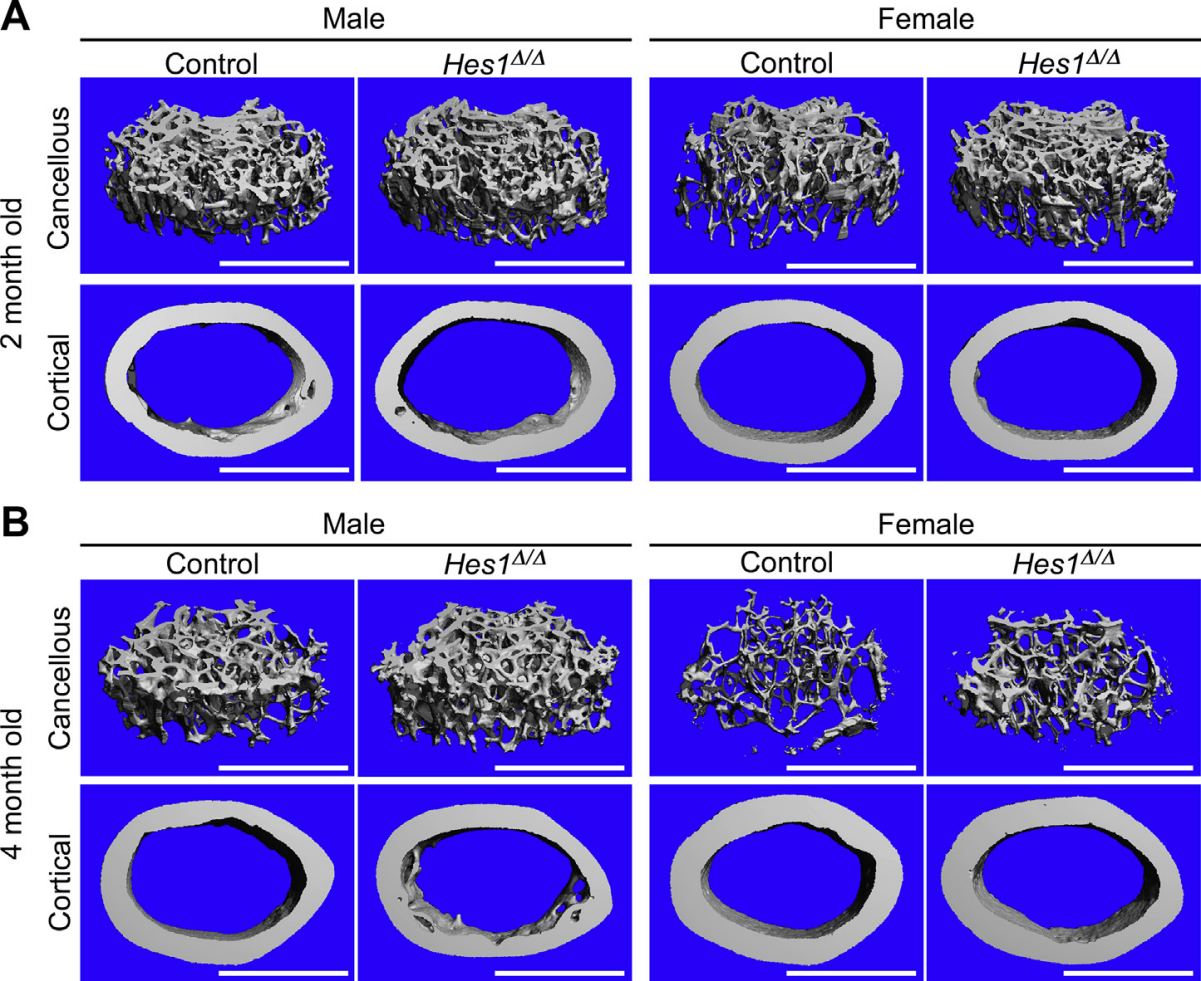
**Inactivation of *Hes1* in *Ctsk*-expressing cells increases bone volume in female mice.** Representative microcomputed tomography image of femurs from 2- (*A*) and 4-month-old (*B*) male and female *Ctsk*^*Cre/WT*^*;Hes1*^*Δ/Δ*^ mice and *Hes1*^*loxP/loxP*^ sex-matched control littermates. The scale bar in the *right corner* represents 1 mm.

**Figure 5:**
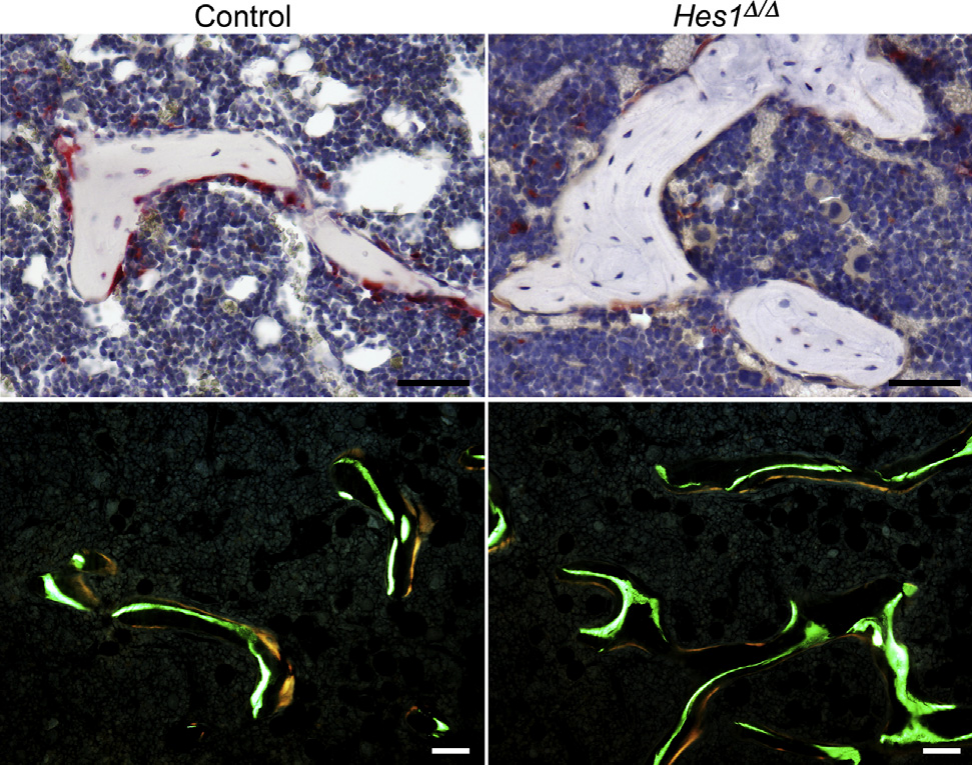
**Inactivation of *Hes1* in *Ctsk*-expressing cells decreases osteoclast number and bone resorption *in vivo*.** Representative static (*upper panels*) and dynamic (*lower panels*) cancellous bone histomorphometry of the distal femur from 4-month-old *Ctsk*^*Cre/WT*^*;Hes1*^*Δ/Δ*^ female mice and *Hes1*^*loxP/loxP*^ sex-matched control littermates. The scale bar in the *right corner* represents 50 μm.

**Supplemental Figure S2:**
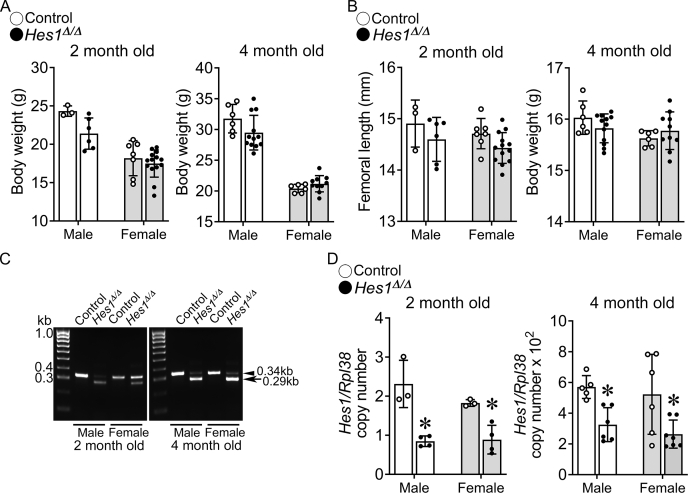


To confirm a role of HES1 in osteoclastogenesis and bone homeostasis, *Hes1* was inactivated *in vivo* in *Ctsk*-expressing cells. For this purpose, *Ctsk*^*Cre/WT*^*;Hes1*^*loxP/loxP*^ mice were crossed with *Hes1*^*loxP/loxP*^ mice to generate *Ctsk*^*Cre/WT*^*;Hes1*^*Δ/Δ*^ and littermate *Hes1*^*loxP/loxP*^ controls. *Ctsk*^*Cre/WT*^*;Hes1*^*Δ/Δ*^ appeared healthy, and their weight and femoral length were not different from littermate *Hes1*^*loxP/loxP*^ mice (Fig. S2)*. Ctsk*^*Cre*^-mediated recombination was documented in genomic DNA from tibiae of *Ctsk*^*Cre/WT*^*;Hes1*^*Δ/Δ*^ mice with a consequent decrease in *Hes1* mRNA. Confirming the results observed in the context of the *Notch2*^*tm1.1Ecan*^ mutant mice, inactivation of *Hes1* in 2- or 4-month-old male mice did not result in an obvious skeletal phenotype, although trabecular number and connectivity were modestly increased (Table 2). In contrast, 2-month and particularly 4-month-old female mice harboring the inactivation of *Hes1* exhibited a significant increase in femoral BV/TV (Table 2 and Fig. 4). Femoral μCT of 4-month-old female *Ctsk*^*Cre/WT*^*;Hes1*^*Δ/Δ*^ mice revealed an 85% increase in BV/TV associated with an increase in trabecular number and connectivity density and a decrease in structure model index (SMI) compared with controls. Bone histomorphometry of 4-month-old *Ctsk*^*Cre/WT*^*;Hes1*^*Δ/Δ*^ female mice demonstrated an ∼50% decrease in osteoclast number and ∼35% decrease in eroded surface, compared with littermate controls, confirming that HES1 is required for osteoclast differentiation and function *in vivo* (Table 3 and Fig. 5). Osteoblast number and bone formation were not affected by the *Hes1* deletion.

**Table 2:** Femoral microarchitecture assessed by μCT of 2- and 4-month-old *Ctsk*^*Cre/WT*^*;Hes1*^*Δ/Δ*^ mice and sex-matched littermate controls

Two months old	Males		Females	
	Control	*Hes1*^*Δ/Δ*^	Control	*Hes1*^*Δ/Δ*^
	n = 3	n = 6	n = 6–7	n = 14
Distal femur trabecular bone				
BV/TV (%)	19.3 ± 3.8	20.4 ± 3.3	10.4 ± 1.4	13.0 ± 1.0^a^
Trabecular separation (μm)	150 ± 9	140 ± 8	206 ± 11	180 ± 10^a^
Trabecular number (1/mm)	6.6 ± 0.6	7.3 ± 0.3^a^	5.0 ± 0.3	5.6 ± 0.3^a^
Trabecular thickness (μm)	44 ± 6	42 ± 2	39 ± 2	39 ± 3
Connectivity density (1/mm^3^)	391 ± 34	474 ± 35^a^	220 ± 37	322 ± 40^a^
Structure model index	1.7 ± 0.3	1.6 ± 0.4	2.3 ± 0.3	2.2 ± 0.2
Density of material (mg HA/cm^3^)	849 ± 28	797 ± 50	814 ± 56	801 ± 55
Femoral midshaft cortical bone				
BV/TV (%)	88.6 ± 1.4	88.0 ± 0.4	88.5 ± 0.8	88.1 ± 1.2
Porosity (%)	11.4 ± 1.4	12.0 ± 0.4	11.5 ± 0.8	11.9 ± 1.2
Cortical thickness (μm)	147 ± 13	144 ± 5	141 ± 7	144 ± 12
Total area (mm^2^)	2.0 ± 0.1	2.0 ± 0.2	1.8 ± 0.2	1.8 ± 0.1
Bone area (mm^2^)	0.9 ± 0.1	0.8 ± 0.1	0.7 ± 0.0	0.7 ± 0.1
Periosteal perimeter (mm)	5.1 ± 0.2	5.0 ± 0.2	4.8 ± 0.0	4.7 ± 0.2
Endocortical perimeter (mm)	3.8 ± 0.1	3.8 ± 0.2	3.7 ± 0.1	3.6 ± 0.1
Density of material (mg HA/cm^3^)	1071 ± 45	1040 ± 13	1079 ± 42	1062 ± 42

Four months old	Males		Females	
	Control	*Hes1*^*Δ/Δ*^	Control	*Hes1*^*Δ/Δ*^
	n = 6	n = 11–12	n = 6	n = 10
Distal femur trabecular bone				
BV/TV (%)	15.8 ± 3.0	17.9 ± 1.7	4.6 ± 1.0	8.5 ± 2.8^a^
Trabecular separation (μm)	192 ± 15	174 ± 10^a^	302 ± 27	255 ± 17^a^
Trabecular number (1/mm)	5.2 ± 0.5	5.7 ± 0.3	3.3 ± 0.3	4.0 ± 0.3^a^
Trabecular thickness (μm)	44 ± 2	42 ± 4	37 ± 3	40 ± 7
Connectivity density (1/mm^3^)	212 ± 44	271 ± 40^a^	82 ± 23	158 ± 44^a^
Structure model index	1.4 ± 0.4	1.2 ± 0.2	2.8 ± 0.1	2.2 ± 0.3^a^
Density of material (mg HA/cm^3^)	903 ± 26	908 ± 24	902 ± 27	896 ± 28
Femoral midshaft cortical bone				
BV/TV (%)	91.7 ± 2.0	90.0 ± 2.6	92.0 ± 0.4	91.8 ± 0.6
Porosity (%)	8.3 ± 2.0	10.0 ± 2.6	8.0 ± 0.4	8.2 ± 0.6
Cortical thickness (μm)	182 ± 13	174 ± 10	186 ± 6	184 ± 7
Total area (mm^2^)	2.1 ± 0.2	2.2 ± 0.2	1.7 ± 0.1	1.8 ± 0.1^a^
Bone area (mm^2^)	1.0 ± 0.1	1.0 ± 0.1	0.8 ± 0.0	0.9 ± 0.0^a^
Periosteal perimeter (mm)	5.2 ± 0.2	5.3 ± 0.2	4.6 ± 0.1	4.8 ± 0.1^a^
Endocortical perimeter (mm)	3.7 ± 0.2	3.8 ± 0.2	3.3 ± 0.2	3.4 ± 0.1
Density of material (mg HA/cm^3^)	1168 ± 25	1166 ± 19	1190 ± 37	1193 ± 22

μCT was performed on distal femurs for trabecular bone and midshaft for cortical bone. Values are means ± SD.

a Significantly different from control, *p* < 0.05.

**Table 3:** Cancellous bone histomorphometry of 4-month-old *Ctsk*^*Cre/WT*^*;Hes1*^*Δ/Δ*^ female mice and sex-matched littermate controls

Distal femur trabecular bone	Control	*Hes1*^*Δ/Δ*^
	n = 4–5	n = 6–8
BV/TV (%)	9.3 ± 2.3	13.6 ± 2.2^a^
Trabecular separation (μm)	317 ± 69	229 ± 38^a^
Trabecular number (1/mm)	3.0 ± 0.7	3.9 ± 0.5^a^
Trabecular thickness (μm)	31 ± 3.2	34 ± 4.0
Osteoblast surface/bone surface (%)	15.9 ± 2.2	15.3 ± 2.0
Osteoblasts/bone perimeter (1/mm)	12.4 ± 2.6	12.6 ± 1.6
Osteoclast surface/bone surface (%)	24.4 ± 8.1	12.6 ± 3.5^a^
Osteoclasts/bone perimeter (1/mm)	8.3 ± 2.1	4.7 ± 1.1^a^
Eroded surface/bone surface (%)	16.5 ± 6.5	10.6 ± 2.4^a^
Mineral apposition rate (μm/day)	1.7 ± 0.1	1.8 ± 0.4
Mineralizing surface/bone surface (%)	31.1 ± 5.1	32.8 ± 2.5
Bone formation rate (μm^3^/μm^2^/day)	0.5 ± 0.1	0.6 ± 0.1

Bone histomorphometry was performed on distal femurs from 4-month-old *Ctsk*^*Cre/WT*^*;Hes1*^*Δ/Δ*^ female mice and sex-matched littermate controls. Values are means ± SD.

a Significantly different from control, *p* < 0.05.

### Inactivation of Hes1 decreases osteoclast differentiation *in vitro*

**Figure 6:**
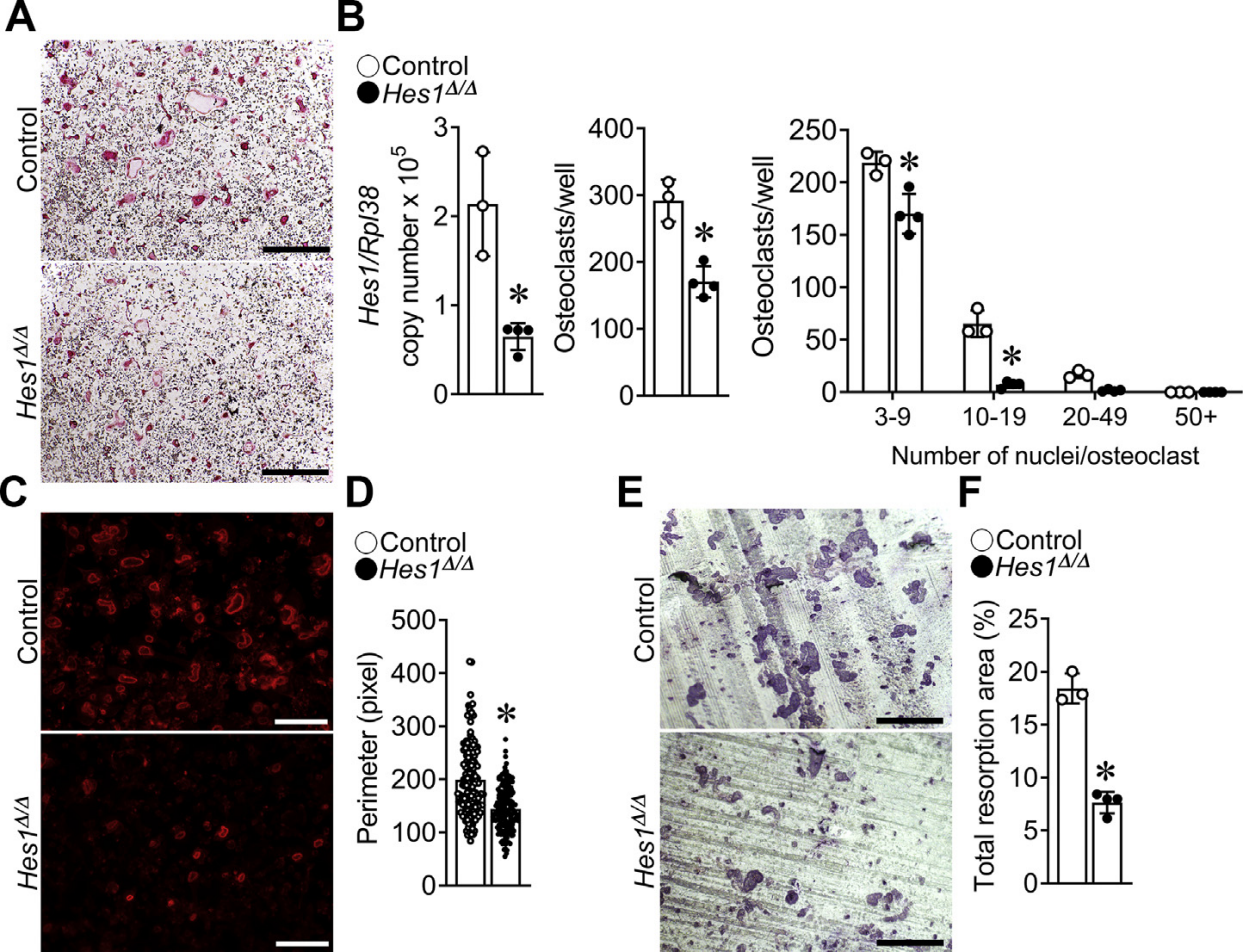
**Number, size, and resorptive capacity are decreased in *Ctsk***^***Cre/WT***^***;Hes1***^***Δ/Δ***^**osteoclasts.** BMMs derived from 2-month-old *Ctsk*^*Cre/WT*^*;Hes1*^*Δ/Δ*^ (*closed circles*) mice and control littermates (*open circles*) were cultured for 4 days in the presence of M-CSF at 30 ng/ml and of RANKL at 10 ng/ml in cell culture–coated plates (*A* and *B*) or bone discs (*C*–*F*). *A*, representative images of TRAP-stained multinucleated cells in cell culture–coated plates are shown. The scale bar in the *right corner* represents 500 μm. *B*, *Hes1* transcript levels were measured by quantitative RT–PCR in total RNA from osteoclasts. Transcript levels are reported as copy number corrected for *Rpl38* (*left*). TRAP-positive cells with more than three nuclei were considered osteoclasts and counted (*middle*). TRAP-positive cells with differential counting of nuclei/osteoclast are shown (*right*). *C*, representative images of Alexa Fluor 594 phalloidin-stained multinucleated cells on bone discs are shown. The scale bar in the *right corner* represents 100 μm. *D*, the perimeter of sealing zones was measured in n = 145 osteoclasts from control and n = 139 osteoclasts from *Ctsk*^*Cre/WT*^*;Hes1*^*Δ/Δ*^ cultures. *E*, representative images of toluidine blue–stained resorption pits. The scale bar in the *right corner* represents 200 μm. *F*, the total resorption pit area was measured (%). Values are means ± SD; n = 3 or 4 biological replicates for control and *Ctsk*^*Cre/WT*^*;Hes1*^*Δ/Δ*^. ∗Significantly different between *Ctsk*^*Cre/WT*^*;Hes1*^*Δ/Δ*^ and control, *p* < 0.05. BMM, bone marrow–derived macrophage; M-CSF, macrophage colony-stimulating factor; RANKL, receptor activator of NF-κB ligand; TRAP, tartrate resistant acid phosphatase.

To confirm that the phenotype of *Ctsk*^*Cre/WT*^*;Hes1*^*Δ/Δ*^ mice was due to a decrease in osteoclast differentiation, BMMs derived from *Ctsk*^*Cre/WT*^*;Hes1*^*Δ/Δ*^ and control littermates were cultured in the presence of M-CSF and RANKL. *Ctsk*^*Cre/WT*^*;Hes1*^*Δ/Δ*^ cultures revealed a 42% decrease in osteoclast number when compared with cells from littermate controls (Fig. 6). The number of osteoclasts with high number of nuclei was decreased in *Ctsk*^*Cre/WT*^*;Hes1*^*Δ/Δ*^ cultures compared with controls, indicating that the size of osteoclasts was reduced because of a decrease in the fusion capacity of *Ctsk*^*Cre/WT*^*;Hes1*^*Δ/Δ*^ cells. Mature osteoclasts have a distinct cytoskeletal structure, namely the sealing zone, a circular actin-rich structure formed by podosomes in a cluster to create a ring that is tightly adherent to the bone matrix for efficient bone resorption (33). Phalloidin staining of osteoclasts from *Ctsk*^*Cre/WT*^*;Hes1*^*Δ/Δ*^ mice cultured on bone slices revealed smaller sealing zones than controls and a ∼30% decrease in the perimeter of the sealing zone (Fig. 6). *Ctsk*^*Cre/WT*^*;Hes1*^*Δ/Δ*^ osteoclasts also exhibited a ∼60% decrease in total bone resorption area, indicating a decrease in osteoclast resorptive activity (Fig. 6).

### Induction of HES1 in osteoclasts causes osteopenia

**Supplemental Figure S3:**
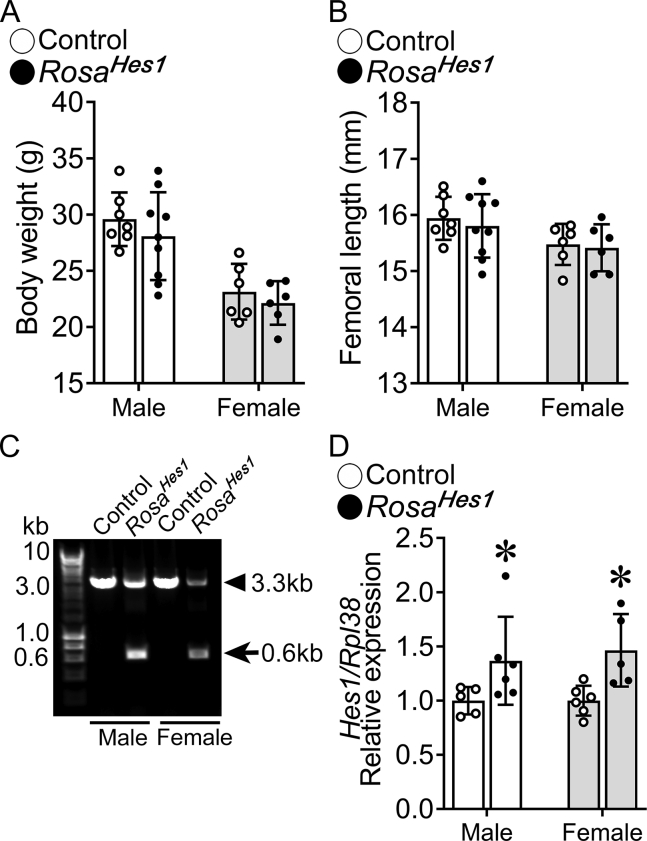


To determine the effect of the HES1 induction on osteoclastogenesis *in vivo*, homozygous *Rosa*^*[STOP]Hes1*^ mice were crossed with *Ctsk*^*Cre/WT*^ mice for the creation of *Ctsk*^*Cre/WT*^*;Rosa*^*Hes1*^ experimental mice and *Rosa*^*[STOP]Hes1*^ littermate controls. *Ctsk*^*Cre/WT*^*;Rosa*^*Hes1*^ mice appeared healthy, and their weight was not different from that of littermate controls (Fig. S3). *Ctsk*^*Cre*^-mediated recombination was demonstrated in genomic DNA from tibiae of *Ctsk*^*Cre/WT*^*;Rosa*^*Hes1*^ mice, and *Hes1* mRNA levels were increased in bone extracts from *Ctsk*^*Cre/WT*^*;Rosa*^*Hes1*^ mice.

**Figure 7:**
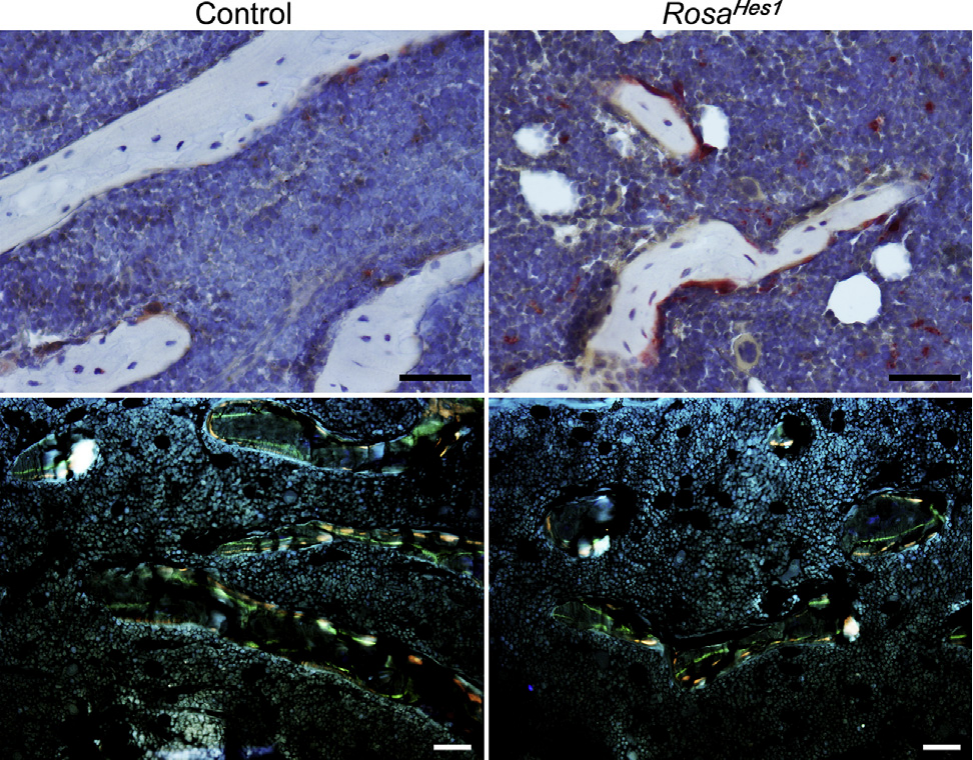
**Activation of HES1 in *Ctsk*-expressing cells increases osteoclast number and bone resorption *in vivo*.** Representative static (*upper panels*) and dynamic (*lower panels*) cancellous bone histomorphometry of the distal femur from 10-week-old *Ctsk*^*Cre/WT*^*;Rosa*^*Hes1*^ female mice and sex-matched littermate controls. The scale bar in the *right corner* represents 50 μm. HES1, hairy and enhancer of split 1.

Femoral architecture of 10-week-old male and female *Ctsk*^*Cre/WT*^*;Rosa*^*Hes1*^ mice revealed a 30% decrease in BV/TV associated with a decrease in connectivity and an increase in SMI in *Ctsk*^*Cre/WT*^*;Rosa*^*Hes1*^ mice that reached statistical significance in female but not in male mice (Table 4). Bone histomorphometry of 10-week-old female *Ctsk*^*Cre/WT*^*;Rosa*^*Hes1*^ mice demonstrated a 1.7-fold increase in osteoclast surface and number, and approximately twofold increase in eroded surface, when compared with littermate controls, confirming that HES1 increases osteoclast differentiation and function *in vivo* (Table 5 and Fig. 7).

**Table 4:** Femoral microarchitecture assessed by μCT of 10-week-old *Ctsk*^*Cre/WT*^*;Rosa*^*Hes1*^ mice and sex-matched littermate controls

μCT parameters	Males		Females	
	Control	*Rosa*^*Hes1*^	Control	*Rosa*^*Hes1*^
	n = 7	n = 9	n = 6	n = 6
Distal femur trabecular bone				
BV/TV (%)	12.5 ± 2.6	10.5 ± 4.1	4.7 ± 0.7	3.3 ± 0.6^a^
Trabecular separation (μm)	206 ± 26	225 ± 25	313 ± 33	346 ± 54
Trabecular number (1/mm)	4.9 ± 0.6	4.5 ± 0.5	3.3 ± 0.3	3.0 ± 0.4
Trabecular thickness (μm)	44 ± 5	42 ± 6	39 ± 3	39 ± 4
Connectivity density (1/mm^3^)	280 ± 82	214 ± 83	92 ± 19	46 ± 18^a^
Structure model index	2.0 ± 0.3	2.1 ± 0.5	2.7 ± 0.2	3.0 ± 0.3^a^
Density of material (mg HA/cm^3^)	980 ± 10	973 ± 9.7	995 ± 9	993 ± 16
Femoral midshaft cortical bone				
BV/TV (%)	89.4 ± 0.1	88.8 ± 0.8	87.3 ± 0.8	87.8 ± 1.1
Porosity (%)	10.6 ± 0.1	11.2 ± 0.8	12.7 ± 0.8	12.2 ± 1.1
Cortical thickness (μm)	191 ± 14	186 ± 17	152 ± 10	159 ± 17
Total area (mm^2^)	2.3 ± 0.2	2.2 ± 0.3	1.8 ± 0.1	1.7 ± 0.1
Bone area (mm^2^)	1.1 ± 0.1	1.1 ± 0.2	0.8 ± 0.1	0.8 ± 0.1
Periosteal perimeter (mm)	5.4 ± 0.3	5.2 ± 0.4	4.7 ± 0.2	4.6 ± 0.2
Endocortical perimeter (mm)	3.9 ± 0.2	3.7 ± 0.2	3.5 ± 0.2	3.4 ± 0.1
Density of material (mg HA/cm^3^)	1181 ± 12	1187 ± 18	1195 ± 14	1195 ± 16

μCT was performed on distal femurs for trabecular bone and midshaft for cortical bone. Values are means ± SD.

a Significantly different from control, *p* < 0.05.

**Table 5:** Cancellous bone histomorphometry of 10-week-old *Ctsk*^*Cre/WT*^*;Rosa*^*Hes1*^ female mice and sex-matched littermate controls

Distal femur trabecular bone	Control	*Rosa*^*Hes1*^
	n = 3–4	n = 3–5
BV/TV (%)	11.2 ± 1.4	8.2 ± 1.6^a^
Trabecular separation (μm)	286 ± 42	383 ± 60^a^
Trabecular number (1/mm)	3.1 ± 0.4	2.4 ± 0.4^a^
Trabecular thickness (μm)	35 ± 3.0	34 ± 2.9
Osteoblast surface/bone surface (%)	17.2 ± 5.6	16.9 ± 3.8
Osteoblasts/bone perimeter (1/mm)	11.3 ± 3.1	12.4 ± 1.5
Osteoclast surface/bone surface (%)	10.8 ± 1.7	18.7 ± 1.4^a^
Osteoclasts/bone perimeter (1/mm)	4.1 ± 0.6	6.8 ± 0.8^a^
Eroded surface/bone surface (%)	12.3 ± 2.8	23.1 ± 4.3^a^
Mineral apposition rate (μm/day)	1.7 ± 0.3	1.4 ± 0.7
Mineralizing surface/bone surface (%)	17.7 ± 7.7	18.7 ± 2.8
Bone formation rate (μm^3^/μm^2^/day)	0.3 ± 0.2	0.3 ± 0.1

Bone histomorphometry was performed on distal femurs from 10-week-old *Ctsk*^*Cre/WT*^*;Rosa*^*Hes1*^ female mice and sex-matched littermate controls. Values are means ± SD.

a Significantly different from control, *p* < 0.05.

### Induction of HES1 enhances osteoclast differentiation *in vitro*

**Figure 8:**
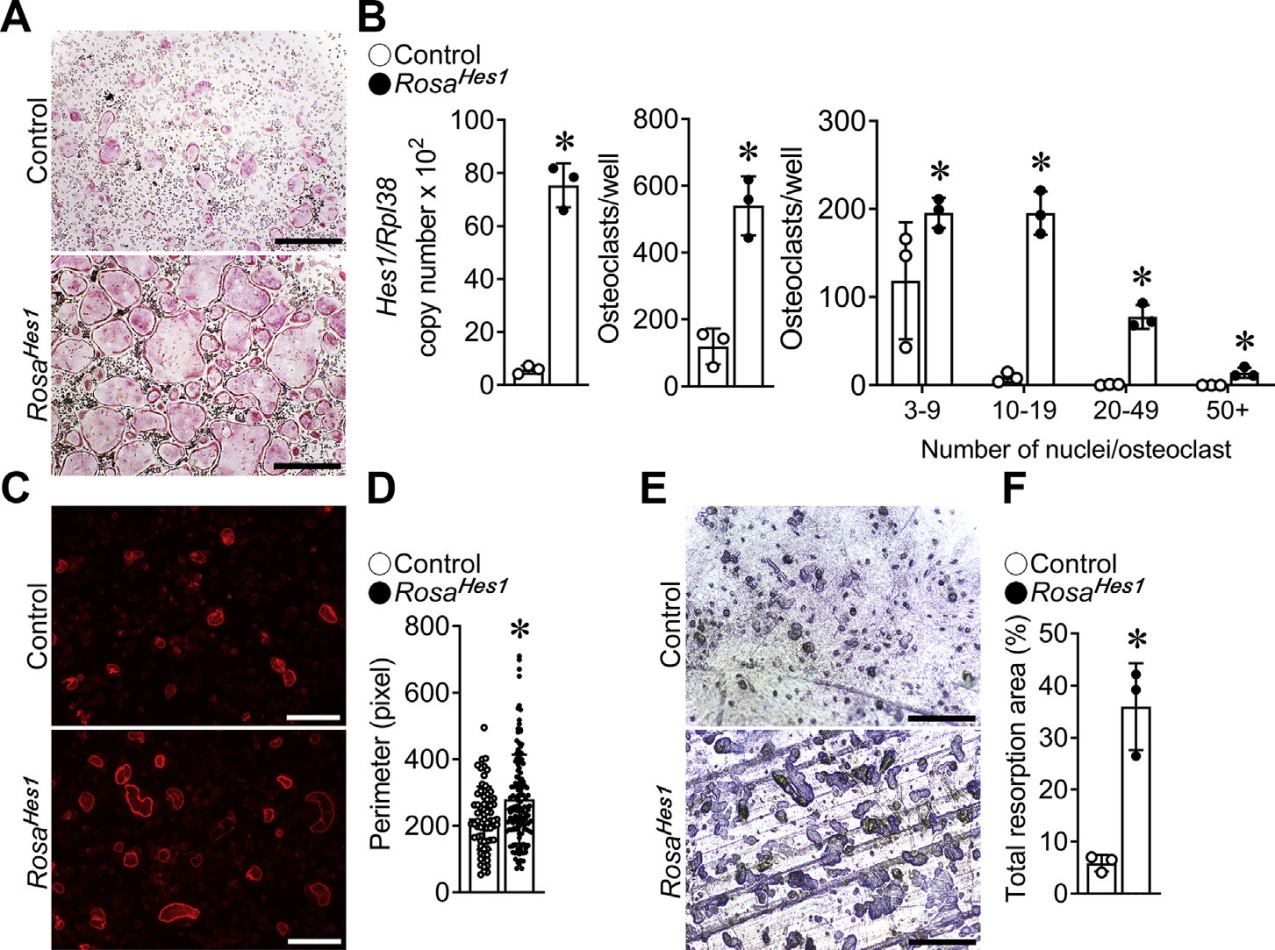
**Number, size, and resorptive capacity are increased in *Ctsk***^***Cre/WT***^***;Rosa***^***Hes1***^**osteoclasts.** BMMs derived from 10-week-old *Ctsk*^*Cre/WT*^*;Rosa*^*Hes1*^ (*closed circles*) mice and control littermates (*open circles*) were cultured for 4 days in the presence of M-CSF at 30 ng/ml and of RANKL at 10 ng/ml in cell culture–coated plates (*A* and *B*) or bone discs (*C*–*F*). *A*, representative images of TRAP-stained multinucleated cells in cell culture–coated plates are shown. The scale bar in the *right corner* represents 500 μm. *B*, *Hes1* transcript levels were measured by quantitative RT–PCR in total RNA from osteoclasts. Transcript levels are reported as copy number corrected for *Rpl38* (*left*). TRAP-positive cells with more than three nuclei were considered osteoclasts and counted (*middle*). TRAP-positive cells with differential counting of nuclei/osteoclast are shown (*right*). *C*, representative images of Alexa Fluor 594 phalloidin-stained multinucleated cells on bone discs are shown. The scale bar in the *right corner* represents 100 μm. *D*, the perimeter of sealing zones was measured in n = 68 osteoclasts from control and in n = 131 osteoclasts from *Ctsk*^*Cre/WT*^*;Rosa*^*Hes1*^ cultures. *E*, representative images of toluidine blue–stained resorption pits. The scale bar in the *right corner* represents 200 μm. *F*, the total resorption pit area was measured (%). Values are means ± SD; n = 3 biological replicates for control and *Ctsk*^*Cre/WT*^*;Rosa*^*Hes1*^. ∗Significantly different between *Ctsk*^*Cre/WT*^*;Rosa*^*Hes1*^ and control, *p* < 0.05. BMM, bone marrow–derived macrophage; M-CSF, macrophage colony-stimulating factor; RANKL, receptor activator of NF-κB ligand; TRAP, tartrate resistant acid phosphatase.

To verify that the phenotype of *Ctsk*^*Cre/WT*^*;Rosa*^*Hes1*^ mice was due to a direct effect in cells of the osteoclast lineage, BMMs from *Ctsk*^*Cre/WT*^*;Rosa*^*Hes1*^ and control littermates were cultured in the presence of M-CSF and RANKL. BMMs from *Ctsk*^*Cre/WT*^*;Rosa*^*Hes1*^ mice exhibited a 4.5-fold increase in osteoclast number in comparison to cells from littermate controls (Fig. 8). In addition, osteoclasts with a high number of nuclei were significantly increased in *Ctsk*^*Cre/WT*^*;Rosa*^*Hes1*^ cultures compared with controls, indicating that the size of osteoclasts was larger because of highly activated fusion in *Ctsk*^*Cre/WT*^*;Rosa*^*Hes1*^ cells. Phalloidin staining of osteoclasts from *Ctsk*^*Cre/WT*^*;Rosa*^*Hes1*^ mice cultured on bone slices confirmed larger cells with sealing zones that were 25% larger than in cells from control littermates (Fig. 8). Accordingly, *Ctsk*^*Cre/WT*^*;Rosa*^*Hes1*^ osteoclasts exhibited a sixfold increase in total resorption pit area (Fig. 8), indicating enhanced bone resorptive capacity in *Ctsk*^*Cre/WT*^*;Rosa*^*Hes1*^ osteoclasts.

### Mechanisms of HES1 action on osteoclastogenesis

**Figure 9:**
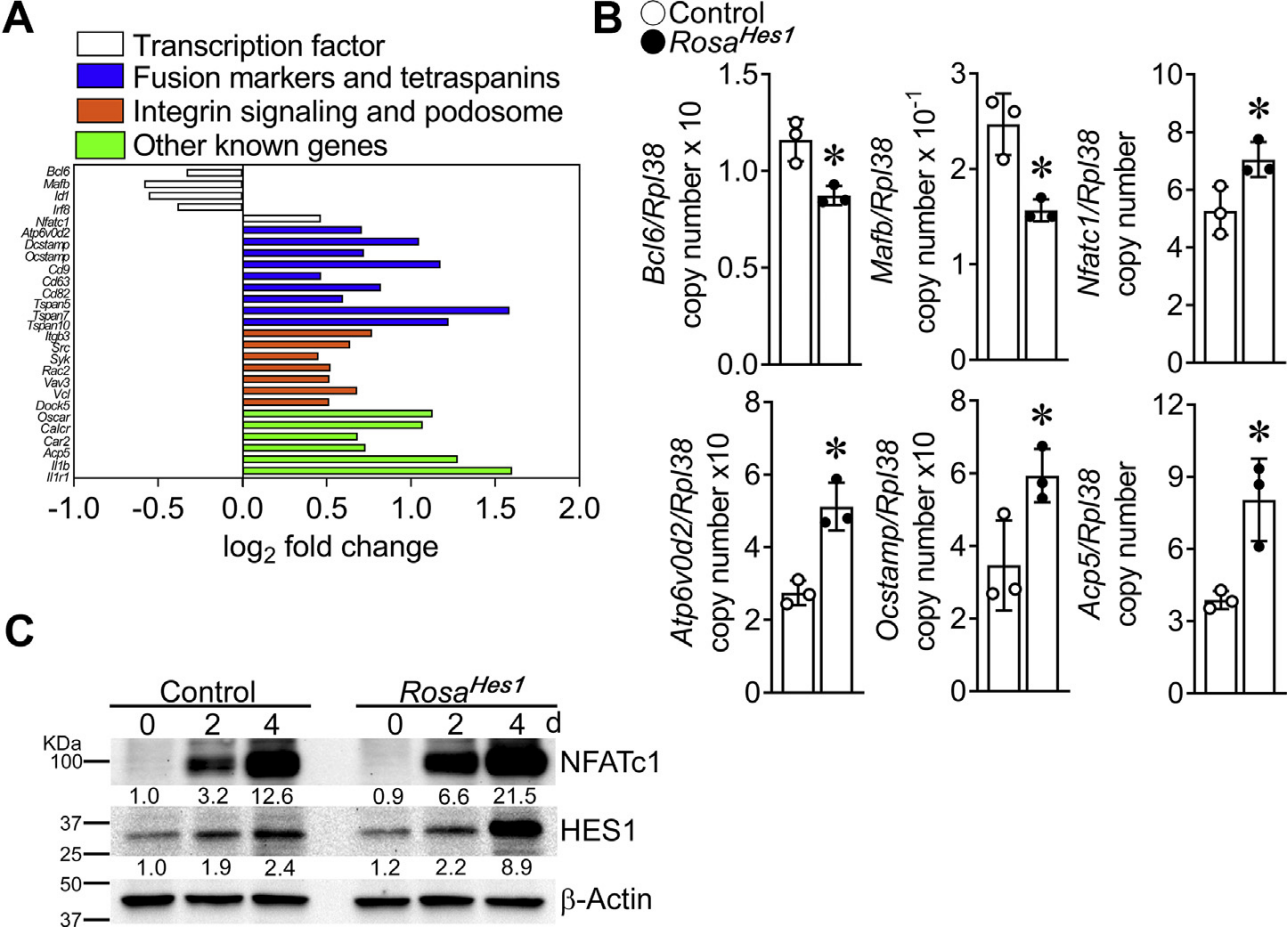
**Expression of osteoclastogenic genes is increased in *Ctsk***^***Cre/WT***^***;Rosa***^***Hes1***^**osteoclasts.** BMMs derived from 10-week-old *Ctsk*^*Cre/WT*^*;Rosa*^*Hes1*^ mice and control littermates were cultured for 4 days in the presence of M-CSF at 30 ng/ml and of RANKL at 10 ng/ml. Cells were collected for total RNA and protein extraction. *A*, RNA was analyzed by RNA-Seq. The *bars* indicate Log_2_ fold changes (*p* < 0.05) of gene expression between control and *Ctsk*^*Cre/WT*^*;Rosa*^*Hes1*^ osteoclasts; n = 3 control and *Ctsk*^*Cre/WT*^*;Rosa*^*Hes1*^ biological replicates. *B*, *Bcl6*, *Mafb*, *Nfatc1*, *Atp6vOd2*, *Ocstamp*, and *Acp* mRNA levels were measured by quantitative RT–PCR and reported as copy number corrected for *Rpl38* mRNA levels. Values are means ± SD; n = 3 control and *Ctsk*^*Cre/WT*^*;Rosa*^*Hes1*^ biological replicates. *C*, representative data of protein levels of NFATc1 and HES1. About 40 μg of total protein were separated by sodium dodecyl sulfate-polyacrylamide gel electrophoresis, and NFATc1 and HES1 levels were detected using anti-NFATc1 and anti-HES1 antibodies, respectively. β-Actin served as a loading control in the same blot. The band intensity was quantified by Image Lab software (version 5.2.1), and the numerical ratio of NFATc1/β-actin and HES1/β-actin is shown under each blot. Control ratios at the initiation of the culture in the presence of RANKL (day 0) are normalized to 1. ∗Significantly different between *Ctsk*^*Cre/WT*^*;Rosa*^*Hes1*^ and control, *p* < 0.05. BMM, bone marrow–derived macrophage; HES1, hairy and enhancer of split 1; M-CSF, macrophage colony-stimulating factor; NFATc1, nuclear factor of activated T cells, cytoplasmic 1; RANKL, receptor activator of NF-κB ligand.

**Supplemental Figure S4:**
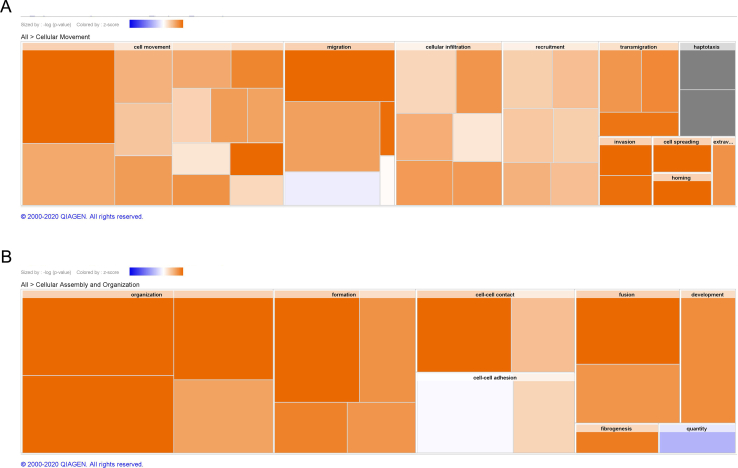


To understand the molecular mechanisms associated with the effect of HES1 on osteoclast differentiation, total RNA from *Ctsk*^*Cre/WT*^*;Rosa*^*Hes1*^ and control osteoclasts was examined by RNA-Seq analysis. Ingenuity pathway analysis (IPA) revealed that ∼200 genes associated with cellular functions, including movement, spreading, cell–cell contact, and organization of the cytoskeleton, were upregulated in cells from *Ctsk*^*Cre/WT*^*;Rosa*^*Hes1*^ mice (Fig. S4). Known fusion markers of osteoclastogenesis, such as *Ocstamp*, *Dcstamp*, and *Atp6v0d2*, were significantly increased in *Ctsk*^*Cre/WT*^*;Rosa*^*Hes1*^ osteoclasts compared with controls (Fig. 9) (34, 35, 36, 37, 38, 39). Tetraspanins, a family of 32 distinct members, are known to affect cell–cell fusion, motility, and sealing zone formation (40, 41, 42). Among the tetraspanins, the transcripts of *Cd9*, *Cd63*, *Cd82*, *Tspan5*, *Tspan7*, and *Tspan10* were expressed in osteoclasts and significantly increased in *Ctsk*^*Cre/WT*^*;Rosa*^*Hes1*^ osteoclasts. Analysis of altered canonical pathway in *Ctsk*^*Cre/WT*^*;Rosa*^*Hes1*^ osteoclasts by IPA revealed upregulation of integrin signaling in *Ctsk*^*Cre/WT*^*;Rosa*^*Hes1*^ osteoclasts (Fig. S5). Osteoclasts express αvβ3 integrins, and they play a role in the adhesion of osteoclasts to bone matrix, cytoskeletal organization, and sealing zone formation (33, 43, 44). Expression of genes associated with integrin signaling, including *itgb3* (integrin β3), *Src*, *Syk*, *Rac2*, *Vav3*, *Vcl* and *Dock5*, was upregulated in *Ctsk*^*Cre/WT*^*;Rosa*^*Hes1*^ osteoclasts (Fig. 9). The transcriptional repressors of *Nfatc1*, including *Bcl6*, *Mafb*, *Id1*, and *Irf8*, were decreased, and *Nfatc1* was increased in *Ctsk*^*Cre/WT*^*;Rosa*^*Hes1*^ osteoclasts; B lymphocyte–induced maturation protein 1 (*Blimp1*) was not affected (45, 46, 47, 48). Interleukin (IL) 1β, known to induce osteoclast differentiation in physiological conditions and following inflammation, and its receptor *Il1r1*, were markedly upregulated in *Ctsk*^*Cre/WT*^*;Rosa*^*Hes1*^ osteoclasts (Fig. 9) (49, 50, 51). The levels of other osteoclastogenic markers, such as *Oscar*, *Calcr*, *Car2*, and *Acp5*, also were increased in *Ctsk*^*Cre/WT*^*;Rosa*^*Hes1*^ osteoclasts. The mRNA expression of *Bcl6*, *Mafb*, *Nfatc1*, *Atp6v0d2*, *Ocstamp*, and *Acp5* was demonstrated by quantitative RT–PCR (qRT–PCR) (Fig. 9). In accordance with these results, NFATc1 protein levels were increased in *Ctsk*^*Cre/WT*^*;Rosa*^*Hes1*^ osteoclasts (Fig. 9). HES1 protein levels were increased in differentiated osteoclasts, and the increase was greater in *Ctsk*^*Cre/WT*^*;Rosa*^*Hes1*^ cells.

## Discussion

The present work uncovers a new function of HES1 on osteoclast differentiation and bone remodeling. The deletion of *Hes1* in *Ctsk*-expressing cells decreased the osteoclastogenic potential of preosteoclasts, whereas its induction enhanced osteoclastogenesis. Osteoclast phenotypes and RNA-Seq analysis revealed that HES1 regulates cell–cell fusion and the formation of the sealing zone. The gene subsets of fusion markers, integrin signaling, and structural proteins for sealing zone formation were significantly upregulated in osteoclasts overexpressing HES1. These results indicate that HES1 has a direct role in osteoclast differentiation and function. Our study also reveals that the expression of *Nfatc1* and that of inhibitors of osteoclastogenesis acting as transcriptional brakes of *Nfatc1*, such as *Irf8*, *Bcl6*, *Mafb*, and *Id1*, were regulated by HES1. It is possible that *HES1* interacts with transcriptional repressors of osteoclastogenesis in a manner analogous to BLIMP1, although the expression of *Blimp1* was not affected by HES1 (47, 52). It is probable that HES1 acts as a transcriptional repressor of inhibitors of osteoclastogenesis and as a consequence causes enhanced *Nfatc1* expression. Under selected cellular conditions, HES1 can act as a transcriptional activator so that one cannot exclude a direct effect of HES1 on the transcriptional activation of *Nfatc1* (30). In accordance with our observations, γ-secretase inhibitors, known to prevent Notch activation, were found to inhibit osteoclast cell fusion and the formation of the podosomal actin belt structure by suppressing HES1/mitogen-activated protein kinase/AKT-mediated induction of NFATc1 *in vitro* (53). However, it is important to note that γ-secretase inhibitors can target many substrates, and their effect is not specific to Notch signaling (54, 55).

Although HES1 had a pronounced effect on osteoclast differentiation and function *in vitro*, this effect was restricted to female mice *in vivo*. The *Hes1* inactivation caused an 85% increase in BV in mature female mice, and the induction of HES1 in *Ctsk*-expressing cells caused an osteopenic phenotype. The inactivation of *Hes1* in male mice did not result in a prominent skeletal phenotype; however, it opposed the osteopenic and resorptive phenotype of *Notch2*^*tm1.1Ecan*^ mice harboring an HCS mutation causing a gain-of-NOTCH2 function. The absence of a phenotype in male mice facilitated the interpretation of the rescue of the *Notch2*^*tm1.1Ecan*^ phenotype by the *Hes1* deletion. Since female *Hes1*-inactivated mice had an increase and *Notch2*^*tm1.1Ecan*^ a decrease in BV, one would expect *Notch2*^*tm1.1Ecan*^*;Hes1*^*Δ/Δ*^ female mice to have an intermediate BV. So that an increase in the BV of *Notch2*^*tm1.1Ecan*^ would not necessarily represent a rescue of the osteopenic phenotype and that HES1 was a mediator of NOTCH2. It is not readily apparent why the *Hes1* inactivation caused a phenotype in female but not in male mice, and the observation stresses the importance of examining phenotypes in mice of different sexes independently (56, 57). It is not unusual to observe sex-specific phenotypes in genetically engineered mice (58, 59, 60). Possible explanations for the prevalence of a phenotype in female mice include genetic influences, a loss of the inhibitory actions of estrogens on osteoclastogenesis in the context of the *Hes1* inactivation as well as the earlier NF-κB–NFATc1 activation and osteoclastogenesis that occurs in female mice (57, 61, 62).

In previous work, we demonstrated that HES1 is induced as osteoclasts mature, particularly in the context of the *Notch2*^*tm1.1Ecan*^ mutation (17). A plausible explanation for the modest skeletal phenotype of the *Hes1* inactivation in male mice is that under basal conditions HES1 levels are low and play a modest role in skeletal physiology, and only following Notch activation, HES1 plays a significant role in bone homeostasis. This explanation is substantiated by the amelioration of the *Notch2*^*tm1.1Ecan*^ osteopenic phenotype following the *Hes1* inactivation. The *Notch2*^*tm1.1Ecan*^ phenotype was not fully reversed, and this is explained by the effects of NOTCH2 enhancing RANKL expression by cells of the osteoblast lineage since these are independent of the induction of HES1 in the myeloid lineage (17, 24). Other Notch target genes, such as *Hey1*, *Hey2*, and *HeyL*, are not expressed in cells of the myeloid lineage and as a consequence could not be responsible for the stimulatory effects of NOTCH2 on osteoclastogenesis (19). HES3 and HES5 could compensate for the effects of HES1, but their expression in osteoclasts is low and their role in osteoclastogenesis is unknown (63). Whereas, HES1 mediates direct effects of NOTCH2 on osteoclastogenesis, it is not likely to mediate the effects of NOTCH1, known to inhibit and not enhance osteoclast maturation, or NOTCH3, since this Notch receptor is not expressed in the myeloid lineage and its effects on osteoclastogenesis are indirect (21, 23). NOTCH4 is expressed at low levels in the myeloid lineage and not known to play a role in osteoclast differentiation (19, 24).

In the present work, we confirm that *Notch2*^*tm1.1Ecan*^ mice are osteopenic because of direct effects of NOTCH2 in cells of the myeloid lineage. The stimulatory effect of NOTCH2 on osteoclastogenesis has been attributed to direct interactions of the NOTCH2 intracellular domain with NF-κB in the context of *Nfatc1* regulatory regions and increased *Nfatc1* transcription (64). However, recent work from our laboratory has demonstrated that NOTCH2 has NF-κB–independent effects on tumor necrosis factor α (TNFα)–induced osteoclastogenesis, and some of these effects are secondary to the activation of AKT and *Il1b* expression (25, 65). The present work demonstrates that the direct effects of NOTCH2 on osteoclastogenesis are HES1 dependent confirming previous work from this laboratory revealing that the enhancement of the osteolytic actions of TNFα by the *Notch2*^*tm1.1Ecan*^ mutation depend on the induction of HES1 (25).

HES1 is known to inhibit phosphatase and tensin homolog and as a consequence enhance phosphoinositide 3-kinase–AKT signaling (66). AKT signaling is required for cell–cell fusion during osteoclast differentiation, and inhibitors of AKT lead to a decrease in *Dcstamp* transcripts and osteoclast size (67). However, the levels of phosphatase and tensin homolog transcripts and the phosphorylation of AKT were not different between *Ctsk*^*Cre/WT*^*;Rosa*^*Hes1*^ osteoclasts and controls (data not shown). Although RANKL and TNFα share and activate similar downstream molecules, mechanisms triggering osteoclastogenesis are different in part because *Nfatc1* and *Dcstamp* levels are not changed in conditions of proinflammatory cytokine-induced osteoclastogenesis (68, 69). *Hes1* inactivation decreases *Il1b* in TNFα-induced *Notch2*^*tm1.1Ecan*^ osteoclasts, and the present work confirms that *Il1b* and *Il1r1* transcripts are increased in HES1-overexpressing osteoclasts (25). IL1β induces pathologically activated osteoclasts bearing a high level of bone-resorbing activity and may be mechanistically relevant to the actions of HES1 in osteoclasts (49).

The phenotype of *Notch2*^*tm1.1Ecan*^ mice as well as the osteopenia of humans harboring HCS pathogenic variants is secondary to an increase in bone resorption with no evidence of impaired bone formation (11, 17, 19). The direct effects of NOTCH2 in the myeloid lineage appear mediated by Notch target gene *Hes1*. This is further substantiated by the fact that other target genes, such as *Hey1*, *Hey2*, and *HeyL*, are not expressed by cells of the osteoclast lineage; therefore, these cannot mediate the effects of NOTCH2 in this cell population.

A limitation of the present work is the use of a *Ctsk*^*Cre*^ mouse model to deliver Cre recombinase since the expression of *Ctsk* is not exclusive to osteoclasts and *Ctsk* is also detected in alternate skeletal and nonskeletal cells (61, 70, 71, 72). Although one cannot fully exclude effects outside the osteoclast lineage, it is reasonable to believe that the effects observed in the present work are secondary to the misexpression of *Hes1* in osteoclasts since cultures of BMMs from *Ctsk*^*Cre/WT*^*;Rosa*^*Hes1*^ and *Ctsk*^*CreWT*^*;Hes1*^*Δ/Δ*^ mice revealed profound effects on osteoclast differentiation. Moreover, the activation and inactivation of *Hes1* in BMM cultures using adenoviruses to deliver Cre demonstrated a direct effect of HES1 in the osteoclast lineage.

In conclusion, HES1 plays a critical role in osteoclastogenesis and bone resorption and is mechanistically relevant to the skeletal phenotype of an experimental model of HCS.

## Experimental procedures

### Genetically modified mice

*Notch2*^*tm1.1Ecan*^ mice harboring a 6955C>T substitution in the *Notch2* locus have been characterized in previous studies and were backcrossed into a C57BL/6 background for eight and more generations (17, 18, 73). *Hes1*^*loxP/loxP*^ (*Hes1<tm1Imayo*) mice, where *loxP* sequences are knocked into the first intron and downstream of the 3′ UTR of *Hes1* alleles, were obtained from RIKEN (RBRC06047; Wako Saitama) in a C57BL/6 background (74). *Rosa*^*[STOP]Hes1*^ (Gt(ROSA)26Sor<tm1(Hes1.EGFP)Imayo>) were obtained from RIKEN (RBRC06002) in an ICR background (26). In *Rosa*^*[STOP]Hes1*^ mice, *Hes1* coding sequences are cloned into the *Rosa26* locus downstream of a Neo-STOP cassette flanked by *loxP* sequences, so that HES1-IRES-GFP is expressed following the excision of the cassette by Cre recombination. To induce or delete *Hes1* in differentiated cells of the osteoclast lineage, mice harboring sequences coding for the Cre recombinase knocked-in into the *Ctsk* locus (*Ctsk*^*Cre*^) were used in a C57BL/6 background (61, 70). Genotyping was conducted in tail DNA extracts by PCR using specific primers from Integrated DNA Technologies (IDT) (Table S1).

For the deletion of *Hes1*, *Hes1*^*loxP*^ alleles were introduced into *Ctsk*^*Cre*^ mice to create *Ctsk*^*Cre/WT*^*;Hes1*^*loxP/loxP*^ mice, and these were crossed with *Hes1*^*loxP/loxP*^ to generate *Hes1*^*Δ/Δ*^-deleted and *Hes1*^*loxP/loxP*^ control littermates. For the induction of HES1, *Ctsk*^*Cre/WT*^ mice were crossed with homozygous *Rosa*^*[STOP]Hes1*^ mice to generate ∼50% *Hes1*-induced and ∼50% *Rosa*^*[STOP]Hes1*^ control littermates. For the deletion of *Hes1* in the context of the *Notch2*^*tm1.1Ecan*^ mutation, *Ctsk*^*Cre/WT*^*;Hes1*^*loxP/loxP*^ mice were crossed with *Notch2*^*tm1.1Ecan*^*;Hes1*^*loxP/loxP*^ mice to create *Notch2*^*tm1.1Ecan*^*;Hes1*^*Δ/Δ*^ and *Notch2*^*tm1.1Ecan*^*;Hes1*^*loxP/loxP*^ controls. Recombination of *loxP* flanked sequences was documented in extracts from tibiae using specific primers (Table S1). All animal experiments were approved by the Institutional Animal Care and Use Committee of UConn Health.

### BMM cultures, osteoclast formation, and adenovirus-Cre-mediated recombination

To obtain BMMs, the marrow from experimental and control sex-matched littermate mice was removed by flushing with a 26-gauge needle, and erythrocytes were lyzed in 150 mM NH_4_Cl, 10 mM KHCO_3_, and 0.1 mM EDTA (pH 7.4), as described previously (73). Cells were centrifuged, and the sediment suspended in α-minimum essential medium (α-MEM) in the presence of 10% fetal bovine serum (FBS; both from Thermo Fisher Scientific) and recombinant human M-CSF at 30 ng/ml. M-CSF complementary DNA (cDNA) and expression vector were obtained from D. Fremont, and M-CSF was purified as previously reported (34). Cells were seeded on uncoated plastic petri dishes at a density of 300,000 cells/cm^2^ and cultured for 3 days. For osteoclast formation, cells were collected following treatment with 0.25% trypsin/EDTA for 5 min and seeded on tissue culture plates at a density of 62,500 cells/cm^2^ in α-MEM with 10% FBS, M-CSF at 30 ng/ml, and recombinant murine RANKL at 10 ng/ml. *Tnfsf11*, encoding RANKL, cDNA expression vector was obtained from M. Glogauer, and glutathione-*S*-transferase–tagged RANKL was expressed and purified as described (75). Cultures were carried out until multinucleated tartrate resistant acid phosphatase (TRAP)–positive cells were formed. TRAP enzyme histochemistry was conducted using a commercial kit (Sigma–Aldrich), in accordance with the manufacturer's instructions. TRAP-positive cells containing more than three nuclei were considered osteoclasts.

For actin structure staining and bone resorption assay of osteoclasts *in vitro*, BMMs were seeded at a density of 62,500 cells/cm^2^ on bovine cortical bone slices and cultured in α-MEM with 10% FBS, M-CSF at 30 ng/ml, and RANKL at 10 ng/ml. To visualize the sealing zone of osteoclasts on bone slices, cells were fixed with 4% paraformaldehyde for 10 min and permeabilized with 0.3% Triton X-100 for 5 min. To block nonspecific background staining, cells on bone discs were incubated with 2% bovine serum albumin for 1 h and stained with Alexa Fluor 594 Phalloidin (Thermo Fisher Scientific) at a 1:40 dilution for 20 min. The sealing zone was viewed on a Leica fluorescence microscope (model DMI6000B), and collected images were processed using the Leica Application Suite X 1.5.1.1387 (Leica Microsystems). After visualizing the sealing zone, cells were stained for TRAP to assess cellular morphology. To visualize bone resorption pits, bone slices were sonicated to remove osteoclasts and stained with 1% toluidine blue in 1% sodium borate. To assess the ability of osteoclasts to resorb bone, the total resorption area/total bone area was measured on images acquired with an Olympus DP72 camera using cellSens Dimension software, version 1.6 (Olympus Corporation). The total resorption area/total bone area was corrected for the total number of TRAP-positive multinucleated cells (73).

To inactivate or induce *Hes1* in osteoclast precursors *in vitro*, BMMs from homozygous *Hes1*^*loxP/loxP*^ or *Rosa*^*[STOP]Hes1*^ mice were cultured in the presence of M-CSF at 30 ng/ml and RANKL at 10 ng/ml for 2 days, prior to being transduced with Ad-Cre or CMV-GFP (Ad-GFP [Vector Biolabs]) as control, at multiplicity of infection of 100 and cultured with M-CSF and RANKL for two additional days until the formation of multinucleated TRAP-positive cells. To inactivate *Hes1* in the context of the *Notch2*^*tm1.1Ecan*^ mutation, *Hes1*^*loxP/loxP*^ alleles were introduced into *Notch2*^*tm1.1Ecan*^ mice to create *Notch2*^*tm1.1Ecan*^*;Hes1*^*loxP/loxP*^ mice, and BMMs were cultured and transduced with Ad-Cre or Ad-GFP.

### qRT–PCR

Total RNA was extracted from osteoclasts with the RNeasy Mini kit (Qiagen) and homogenized bones with the RNeasy Micro kit (Qiagen), in accordance with the manufacturer's instructions. The integrity of the RNA extracted from bones was assessed by microfluidic electrophoresis on an Experion system (Bio-Rad), and RNA with a quality indicator number equal to or higher than 7.0 was used for subsequent analysis. Equal amounts of RNA were reverse transcribed using the iScript RT-PCR kit (Bio-Rad) and amplified in the presence of specific primers (all from IDT; Table S2) with the SsoAdvanced Universal SYBR Green Supermix (Bio-Rad) at 60 °C for 40 cycles. Transcript copy number was estimated by comparison with a serial dilution of cDNA for *Acp5* and *Notch2* (all from Thermo Fisher Scientific), *Hes1* (American Type Culture Collection), *Nfatc1* (Addgene; plasmid 11793; created by A. Rao), *Bcl6*, *Mafb*, *Atp6vOd2*, and *Ocstamp* (all from Dharmacon).

The level of *Notch2*^*6955C>T*^ mutant transcript was measured as described previously (17). Total RNA was reverse transcribed with Moloney murine leukemia virus reverse transcriptase in the presence of reverse primers for *Notch2* (5′-GGATCTGGTACATAGAG-3′) and *Rpl38* (Table S2). *Notch2* cDNA was amplified by qPCR in the presence of TaqMan gene expression assay mix, including specific primers (5′-CATCGTGACTTTCCA-3′ and 5′-GGATCTGGTACATAGAG-3′) and a 6-carboxyfluorexcein-labeled DNA probe of sequence 5′-CATTGCCTAGGCAGC-3′ covalently attached to a 3′-minor groove binder quencher (Thermo Fisher Scientific), and SsoAdvanced Universal Probes Supermix (Bio-Rad) at 60 °C for 45 cycles (76). *Notch2*^*6955C>T*^ transcript copy number was estimated by comparison with a serial dilution of a synthetic DNA fragment (IDT) containing ∼200 bp surrounding the 6955C>T mutation in the *Notch2* locus and cloned into pcDNA3.1(−) (Thermo Fisher Scientific) by isothermal single reaction assembly using commercially available reagents (New England Biolabs) (77).

Amplification reactions were conducted in CFX96 qRT–PCR detection systems (Bio-Rad), and fluorescence was monitored at the end of the elongation step during every PCR cycle. Data are expressed as copy number corrected for *Rpl38* expression estimated by comparison with a serial dilution of cDNA for *Rpl38* (American Type Culture Collection) (78).

### Illumina transcriptome library preparation and sequencing

Total RNA was quantified, and purity ratios were determined for each sample using a NanoDrop 2000 spectrophotometer (Thermo Fisher Scientific). To assess RNA quality, total RNA was analyzed on the Agilent TapeStation 4200 (Agilent Technologies) using the RNA High Sensitivity assay. Ribosomal integrity numbers were recorded for each sample. Only samples with ribosomal integrity number values above 9.0 were used for library preparation.

Total RNA samples were prepared for mRNA-Seq using the Illumina TruSeq Stranded mRNA Sample Preparation kit following the manufacturer's protocol (Illumina). Libraries were validated for length and adapter dimer removal using the Agilent TapeStation 4200 D1000 High Sensitivity assay (Agilent Technologies), and then they were quantified and normalized using the dsDNA High Sensitivity Assay for Qubit 3.0 (Thermo Fisher Scientific). Sample libraries were prepared for Illumina sequencing by denaturing and diluting the libraries per manufacturer's protocol (Illumina). All samples were pooled into one sequencing pool, equally normalized, and run as one sample pool across the Illumina NextSeq 500 using version 2.5 chemistry. Target read depth was achieved for each sample with paired end 75 bp reads. Raw reads were trimmed with Sickle (version 1.33), with a quality threshold of 30 and length threshold of 45, following that the trimmed reads were mapped to Homo Sapiens genome (GRCh38 ensembl release 99) with HISAT2 (version 2.1.0) (79). The resulting SAM files were then converted into BAM format using samtools (version 1.9) (80), and the PCR duplicates were removed using PICARD (http://broadinstitute.github.io/picard/). The counts were generated against the features with HTSeq-count (81). The differential expression of genes between conditions was evaluated using DESeq2 (82). Covariates were introduced in the DESeq2 analysis to increase the accuracy of results, and genes showing less than ten counts across the compared samples were excluded from the analysis. Genes with a false discovery rate <0.05 were considered significant and used in the downstream analysis. The processed RNA-Seq results were further analyzed by using IPA (Qiagen).

### Immunoblotting

Cells from control and experimental mice were extracted in buffer containing 25 mM Tris–HCl (pH 7.5), 150 mM NaCl, 5% glycerol, 1 mM EDTA, 0.5% Triton X-100, 1 mM sodium orthovanadate, 10 mM NaF, 1 mM phenyl methyl sulfonyl fluoride, and a protease inhibitor cocktail (all from Sigma–Aldrich). Total cell lysates (40 μg of total protein) were separated by sodium dodecyl sulfate-polyacrylamide gel electrophoresis in 8 or 12% polyacrylamide gels and transferred to Immobilon-P membranes (Millipore). The blots were probed with anti-HES1 (11988) and β-actin (3700) antibodies from Cell Signaling Technology or anti-NFATc1 antibody (556602) from BD Biosciences. The blots were exposed to anti-rabbit, antirat, or antimouse IgG conjugated to horseradish peroxidase (Sigma–Aldrich) and incubated with a chemiluminescence detection reagent (Bio-Rad). Chemiluminescence was detected by ChemiDoc XSR+ molecular imager (Bio-Rad) with Image Lab software (version 5.2.1), and the amount of protein present in individual bands was quantified (25).

### μCT

Femoral microarchitecture was determined using a μCT instrument (Scanco μCT 40; Scanco Medical AG), which was calibrated periodically using a phantom provided by the manufacturer (83, 84). Femurs were scanned in 70% ethanol at high resolution, energy level of 55 kVp, intensity of 145 μA, and integration time of 200 ms. Evaluation of skeletal microarchitecture was started 1.0 mm proximal from the condyles of the distal femur. A total of 160 consecutive 6 μm thick slices were acquired at an isotropic voxel dimension of 216 μm^3^ and selected for analysis. Contours were drawn manually every ten slices a few voxels away from the endocortical boundary to define the region of analysis. The remaining slice contours were iterated automatically. BV/TV, trabecular separation, number and thickness, connectivity density, SMI, and material density were measured in trabecular regions using a Gaussian filter (σ = 0.8) (83, 84). For analysis of cortical bone, contours were iterated across 100 slices along the cortical shell of the femoral midshaft, excluding the marrow cavity. Analyses of BV/TV, cortical thickness, periosteal perimeter, endosteal perimeter, total cross-sectional area, and cortical bone area were conducted using a Gaussian filter (σ = 0.8, support = 1).

### Bone histomorphometry

Bone histomorphometry was carried out in *Ctsk*^*Cre/WT*^*;Notch2*^*tm1.1Ecan*^*;Hes1*^*Δ/Δ*^, *Ctsk*^*Cre/WT*^*;Hes1*^*Δ/Δ*^, and *Ctsk*^*Cre/WT*^*;Rosa*^*Hes1*^ mice, and sex-matched controls were injected with calcein 20 mg/kg and demeclocycline 50 mg/kg at a 5 or 7 days of interval and sacrificed 2 days after demeclocycline administration. For static cancellous bone histomorphometry and to assess for the presence of TRAP-positive multinucleated cells, bones were decalcified in 14% EDTA for 14 days and embedded in paraffin, and 7 μm sections were stained for the presence of TRAP and counterstained with hematoxylin and analyzed at a 100× magnification using OsteoMeasureXP software (Osteometrics). Stained sections were used to draw bone tissue and measure trabecular separation, number and thickness, and eroded surface, as well as to count osteoblast and osteoclast number. To assess dynamic parameters of bone histomorphometry, undecalcified femurs were embedded in methyl methacrylate, and 5 μm sections were cut using Microm microtome (Richards-Allan Scientific). Mineralizing surface per bone surface and mineral apposition rate were measured on unstained sections visualized under UV light and a triple diamidino-2-phenylindole/fluorescein/Texas red set long-pass filter, and bone formation rate was calculated (85).

### Statistics

Data are expressed as means ± SD and presented as biological replicates except for experiments where BMMs were transduced with adenoviruses or cells were extracted for immunoblotting, and these are presented as technical replicates representative of two or more experiments. Statistical differences were determined by Student's *t* test or two-way analysis of variance with Tukey analysis for multiple comparisons, respectively.

## Data availability

Data not shown will be shared upon request to Ernesto Canalis at canalis@uchc.edu.

## Supporting information

This article contains supporting information.

## Conflict of interest

The authors declare no conflicts of interest with the contents of this article.

## Figures and Tables

**Supplemental Figure S5:**