# Supplementary material for: Hairy and enhancer of split 1 is a primary effector of NOTCH2 signaling and induces osteoclast differentiation and function
Source: J Biol Chem. 2021 Nov 3;297(6):101376. doi: 10.1016/j.jbc.2021.101376 (PMC8633688; doi:10.1016/j.jbc.2021.101376)
Supplement: Supplemental Table S1 [file mmc1.docx]

**Supplementary Table 1.** Primers used for genotyping by PCR.

| **Allele** | **Strand** | **Sequence** | **Amplicon Size (bp)** |
| --- | --- | --- | --- |
| *Ctsk^Cre^* | Forward1 | 5'-GCGGTCTGGCAGTAAAAACTATC-3' | *Ctsk^WT^* = 324  *Ctsk^Cre^* = 100 |
|  | Forward2 | 5'-TAGGCCACAGAATTGAAAGATCT-3' |  |
|  | Reverse1  Reverse2 | 5'-GTGAAACAGCATTGCTGTCACTT-3'  5'-GTAGGTGGAAATTCTAGCATCATCC-3' |  |
| *Rosa^Hes1^* | Forward | 5'-AAAGTCGCTCTGAGTTGTTAT-3' | WT = 603  *Rosa^Hes1^* = 3280 |
|  | Reverse1 | 5'-GGAGCGGGAGAAATGGATATG-3' |  |
|  | Reverse2 | 5'-TGCCCTTCGCCTCTTCTCCATGATA-3' |  |
| *Notch2^tm1.1Ecan^* | Forward | 5'-CCCTTCTCTCTGTGCGGTAG-3' | WT = 308  *Notch2^tm1.1Ecan^* = 403 |
|  | Reverse | 5'-CTCAGAGCCAAAGCCTCACTG-3' |  |
| *Hes1^loxP/loxP^* | Forward | 5'-CAGCCAGTGTCAACACGACACCGGACAAAC-3' | WT = 200  *Hes1^loxP^* = 250 |
|  | Reverse | 5'-TCGCCTTCGCCTCTTCTCCATGATA-3' |  |
| ***LoxP* recombination** | **Strand** | **Sequence** | **Amplicon Size (bp)** |
| *LoxP* recombination of *Rosa^(STOP)Hes1^* | Forward | 5'-AGCACAATGCCCATGTTCAC-3' | Recombined = 603  Not recombined = 3280 |
|  | Reverse | 5'-GGTGTCTCCCTCAAATGTTCA-3' |  |
| *LoxP* recombination of *Hes1^loxP/loxP^* | Forward | 5'- CAGCCAGTGTCAACACGACACCGGACAAAC-3' | Recombined = 291  Not recombined = 342 |
|  | Reverse | 5'- GGTGGGGCTTGAAATTCATGTAGTTTGGC-3' |  |
