# Supplementary material for: Hairy and enhancer of split 1 is a primary effector of NOTCH2 signaling and induces osteoclast differentiation and function
Source: J Biol Chem. 2021 Nov 3;297(6):101376. doi: 10.1016/j.jbc.2021.101376 (PMC8633688; doi:10.1016/j.jbc.2021.101376)
Supplement: Supplemental Table S2 [file mmc2.docx]

| **Gene** | **Strand** | **Sequence** | **GenBank Accession Number** |
| --- | --- | --- | --- |
| *Acp5* | Forward | 5'-GACAAGAGGTTCCAGGAGAC-3' | NM_001102404; NM_001102405;  NM_007388 |
|  | Reverse | 5'-TTCCAGCCAGCACATACC-3' |  |
| *Atp6v0d2* | Forward | 5'-AGAGTTTGACTATTTCCG-3' | NM_175406 |
|  | Reverse | 5'-ATGTCATGTAGGTGAGAA-3' |  |
| *Bcl6* | Forward | 5'-GGCAAGTCCCTAATGAGTATAGC-3' | NM_001348026  NM_009744 |
|  | Reverse | 5'-TCCTTGGGCGAGTAGATGT-3' |  |
| *Hes1* | Forward | 5'-ACCAAAGACGGCCTCTGAGCACAGAAAGT-3' | NM_008235 |
|  | Reverse | 5'-ATTCTTGCCCTTCGCCTCTT-3' |  |
| *Mafb* | Forward | 5'-CAGGAGCAGGTGTGACTC-3' | NM_010658 |
|  | Reverse | 5'-TTGGTGATGATGGTGATGGT-3' |  |
| *Nfatc1* | Forward | 5'-GCGCAAGTACAGTCTCAATGGCC-3' | NM_198429; NM_001164110;  NM_001164111; |
|  | Reverse | 5'-GGATGGTGTGGGTGAGTGGT-3' | NM_001164112;  NM_00116641091;  NM_016791 |
| *Ocstamp* | Forward | 5'-TCACAGTCAAATATGACGCCTC-3' | NM_029021 |
|  | Reverse | 5'-TGAGGACGAAGAGGATGAAGT-3' |  |
| *Notch2* | Forward | 5'-TGACGTTGATGAGTGTATCTCCAAGCC-3' | NM_010928 |
|  | Reverse | 5'-GTAGCTGCCCTGAGTGTTGTGG-3' |  |
| *Rpl38* | Forward | 5'-AGAACAAGGATAATGTGAAGTTCAAGGTTC-3' | NM_001048057; NM_001048058;  NM_023372 |
|  | Reverse | 5'-CTGCTTCAGCTTCTCTGCCTTT-3' |  |

**Supplementary Table 2.** Primers used for qRT-PCR determinations. GenBank accession numbers identify transcript recognized by primer pairs.
