# Supplementary material for: Hairy and enhancer of split 1 is a primary effector of NOTCH2 signaling and induces osteoclast differentiation and function
Source: J Biol Chem. 2021 Nov 3;297(6):101376. doi: 10.1016/j.jbc.2021.101376 (PMC8633688; doi:10.1016/j.jbc.2021.101376)
Supplement: Supplemental Figures Legend [file mmc3.docx]

**Supplementary Figure 1. Inactivation of *Hes1* in *Ctsk*-expressing cells of Hajdu Cheney mutant mice.** Weight, femoral length, documentation of DNA recombination of *loxP* flanked sequences by Cre and *Hes1* copy number in bone extracts. (A and B) Body weight and femoral length of 2 month old *Ctsk^Cre/WT^; Hes1^∆/∆^*  (open circles/grey bar) or *Notch2^tm1.1Ecan^;* *Ctsk^Cre/WT^; Hes1^∆/∆^* (closed circles/grey bar) male mice and sex-matched control *Hes1^loxP/loxP^* (open circles/white bar) or *Notch2^tm1.1Ecan^; Hes1^loxP/loxP^* (closed circles/white bar) littermates. Values are means ± SD; n = 12 for control *Hes1^loxP/loxP^* and n = 6 for *Notch2^tm1.1Ecan^; Hes1^loxP/loxP^*; n = 11 for *Ctsk^Cre/WT^; Hes1^∆/∆^* and n = 12 for *Notch2^tm1.1Ecan^;* *Ctsk^Cre/WT^; Hes1^∆/∆^*. (C) *Hes1, Notch2* and *Notch2^6955C>T^* transcript levels were measured by qRT-PCR in total RNA from tibiae of *Ctsk^Cre/WT^; Hes1^∆/∆^*  or *Notch2^tm1.1Ecan^;* *Ctsk^Cre/WT^; Hes1^∆/∆^* and controls. Transcript levels are reported as copy number corrected for *Rpl38*. Value are means ± SD; n = 6 for control *Hes1^loxP/loxP^* and n = 5 for *Notch2^tm1.1Ecan^; Hes1^loxP/loxP^*; n = 7-8 for *Ctsk^Cre/WT^; Hes1^∆/∆^*  and n = 9 for *Notch2^tm1.1Ecan^;* *Ctsk^Cre/WT^; Hes1^∆/∆^*. *Significantly different between *Notch2^tm1.1Ecan^* and wild type control, *p* < 0.05. #Significantly different between *Hes1^∆/∆^* and *Hes1^loxP/loxP^*, *p* < 0.05.

**Supplementary Figure 2. Inactivation of *Hes1* in *Ctsk*-expressing cells.** Weight, femoral length, documentation of DNA recombination of the *loxP* flanked sequences by Cre and *Hes1* copy number in bone extracts. (A and B) Body weight and femoral length of 2 and 4 month old *Ctsk^Cre/WT^;Hes1^∆/∆^* (closed circles) and sex-matched control *Hes1^loxP/loxP^* (open circles) littermates. Values are means ± SD; n = 3 (2 month) and n = 6 (4 month) for male and n = 7 (2 month) and n = 6 (4 month) female *Hes1^loxP/loxP^* and n = 6 (2 month) and n = 12 (4 month) for male and n = 14 (2 month) and n = 10 (4 month) for female *Ctsk^Cre/WT^;Hes1^∆/∆^* mice. (C) Genomic DNA from tibiae obtained from 2 and 4 month old *Ctsk^Cre/WT^;Hes1^∆/∆^* male and female mice and respective controls was isolated, and DNA recombination of *loxP* flanked sequences was demonstrated by gel electrophoresis of PCR amplification products obtained with primers for the *Hes1* alleles. The arrowhead indicates the position of the 0.34 kilo-base (kb) amplicon verifying the *Hes1^loxP^* allele, and the arrow indicates a 0.29 kb amplicon verifying the recombined *Hes1^∆^* allele. Representative image of PCR amplification products is shown. (D) *Hes1* transcript levels were measured by qRT-PCR in total RNA from tibiae of 2 and 4 month old *Ctsk^Cre/WT^;Hes1^∆/∆^* (closed circles) and controls (open circles). Transcript levels are reported as copy number corrected for *Rpl38*. Values are means ± SD; n = 3 for control and n = 4 for 2 month old *Ctsk^Cre/WT^;Hes1^∆/∆^* male and female mice; n = 5-6 for control and n = 6-7 for 4 month old *Ctsk^Cre/WT^;Hes1^∆/∆^* male and female mice. *Significantly different between *Ctsk^Cre^; Hes1^∆/∆^* and control, *p* < 0.05.

**Supplementary Figure 3. Activation of HES1 in *Ctsk*-expressing cells.** Weight, femoral length, documentation of DNA recombination of the *loxP* flanked STOP cassette by Cre and *Hes1* copy number in bone extracts. (A and B) Body weight and femoral length of 10 week old *Ctsk^Cre/WT^;Rosa^Hes1^* (closed circles) and sex-matched control *Rosa^[STOP]Hes1^* littermates (open circles). Values are means ± SD; n = 7 control and n = 9 *Ctsk^Cre/WT^;Rosa^Hes1^* for 10 week old male mice; n = 6 control and *Ctsk^Cre/WT^;Rosa^Hes1^* for 10 week old female mice. (C) Genomic DNA from tibiae obtained from 10 week old *Ctsk^Cre/WT^;Rosa^Hes1^* male and female mice and respective controls was isolated. DNA recombination of *loxP* flanked STOP cassette was demonstrated by gel electrophoresis of PCR amplification products obtained with primers for the *Rosa^Hes1^* alleles. The arrowhead indicates the position of the 3.3-kilo base (kb) amplicon verifying the *Rosa^[STOP]Hes1^* allele, and the arrow indicates a 0.6 kb amplicon verifying the recombined *Rosa^Hes1^* allele. Representative image of PCR amplification products is shown. (D) *Hes1* transcript levels were measured by qRT-PCR in total RNA from tibiae of *Ctsk^Cre/WT^;Rosa^Hes1^* (closed circles) and controls (open circles). Transcript levels are reported as relative expression corrected for *Rpl38*. Value are means ± SD; n = 5 control and n = 6 *Ctsk^Cre/WT^;Rosa^Hes1^* for 10 week old male mice; n = 6 control and n = 5 *Ctsk^Cre/WT^;Rosa^Hes1^* for 10 week old female mice.

**Supplementary Figure 4. Expression of gene subsets associated with cell movement and assembly were increased in osteoclasts overexpressing HES1.** BMMs derived from 10 week old *Ctsk^Cre/WT^;Rosa^Hes1^* mice and control littermates were cultured 4 days in the presence of M-CSF at 30ng/ml and RANKL at 10ng/ml. Cells were collected for total RNA and analyzed by RNA-seq and results were further analyzed by using Ingenuity Pathway Analysis. Heat map shows increased expression (orange) gene subsets associated with cell movement (*p*-value range, 1.43E-04 - 6.07E-16, 201 molecules) (A) and with cellular assembly and organization (*p*-value range, 1.06E-04 - 5.25E-11, 191 molecules) (B). The heat map intensity indicates activation z-score.

**Supplementary Figure 5. Enhanced Integrin signaling is predicted in osteoclasts overexpressing HES1.** BMMs derived from 10 week old *Ctsk^Cre/WT^;Rosa^Hes1^* mice and control littermates were cultured 4 days in the presence of M-CSF at 30ng/ml and of RANKL at 10ng/ml. Cells were collected for total RNA and analyzed by RNA-seq. The results were further analyzed by using Ingenuity Pathway Analysis. Genes significantly upregulated in HES1 overexpressing osteoclasts are highlighted with a bold purple line and filled pink circles.
